# Supplementary material for: Scaling relation between genome length and particle size of viruses provides insights into viral life history
Source: iScience. 2021 Apr 21;24(5):102452. doi: 10.1016/j.isci.2021.102452 (PMC8169800; doi:10.1016/j.isci.2021.102452)
Supplement: Document S1. Figures S1–S3 and Table S2 [file mmc1.pdf]

iScience, Volume *24*

Supplemental information

Scaling relation between genome length and particle size of viruses provides insights into viral life history

Harshali V. Chaudhari, Mandar M. Inamdar, and Kiran Kondabagil

Supplemental Information

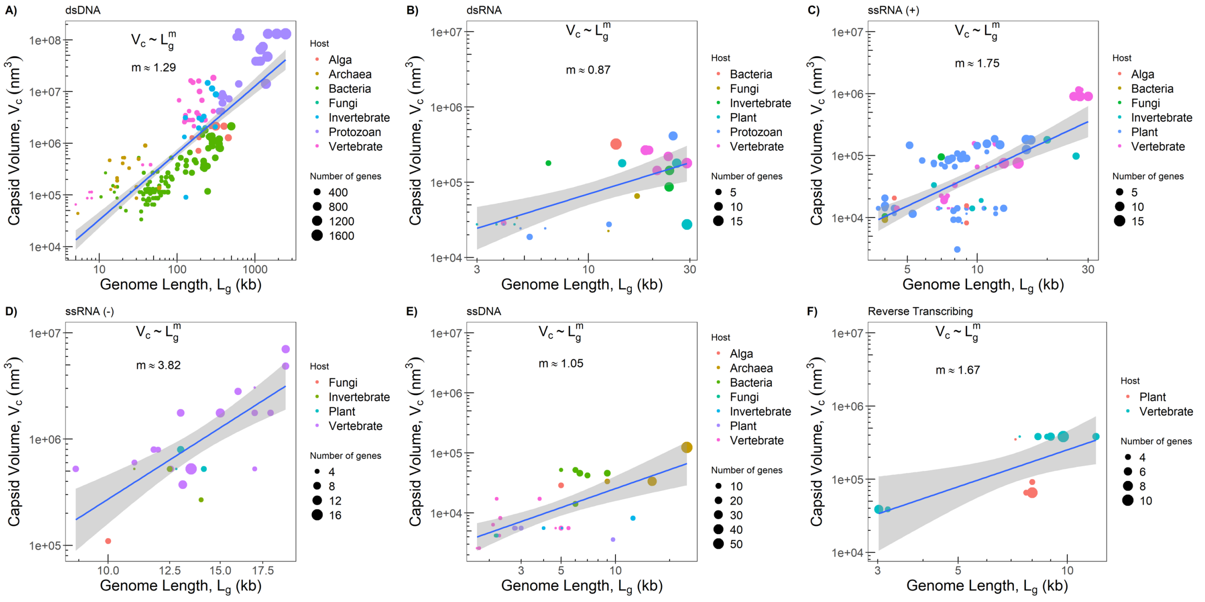

Figure S1. The log-log plot of outer capsid volume as a function of genome length for viruses infecting different hosts according to the Baltimore classification. Related to Figure 1 and STAR Methods "Data analysis".

Size of the data points indicate the number of genes. A power law $y = ax^m$ is used as a fitting expression for the entire data. A linear regression fit of the form $Y = m\,X + A$ to the data, where $Y = log\,y$, $X = log\,x$, and $A = log\,a$, gives

(A) $m = 1.29$ for dsDNA viruses

(B) $m = 0.87$ for dsRNA viruses

(C) $m = 1.75$ for ssRNA (+) positive sense viruses

(D) $m = 3.82$ for ssRNA (-) negative sense viruses

(E) $m = 1.05$ for ssDNA viruses

(F) $m = 1.67$ for reverse transcribing viruses.

All logs are to the base 10. Data and formulas are presented in the Table S1.

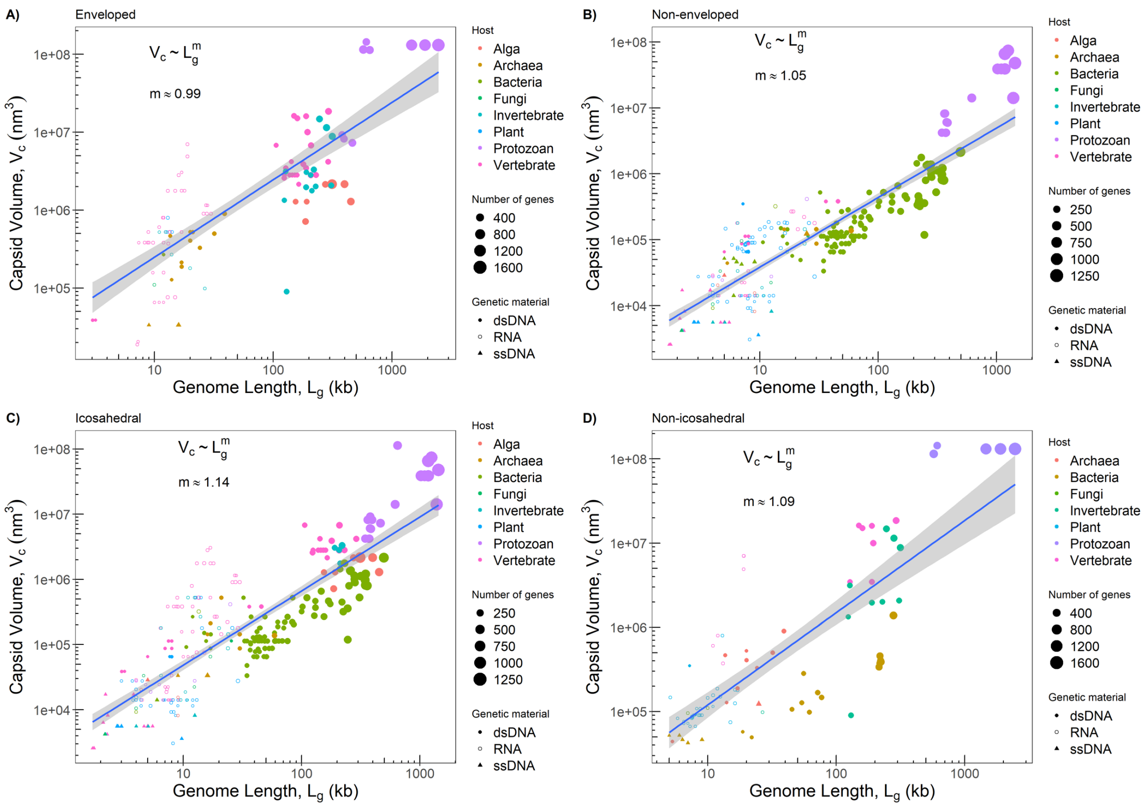

Figure S2. The log-log plot of outer capsid volume as a function of genome length for viruses infecting different hosts according to enveloped (A), non-enveloped (B), icosahedral (C), and non-icosahedral (D) viruses. Related to Figure 1 and STAR Methods “Data analysis”.

Size of the data points indicate the number of genes. A power law $y = ax^m$ is used as a fitting expression for the entire data. A linear regression fit of the form $Y = m\,X + A$ to the data, where $Y = \log y$, $X = \log x$, and $A = \log a$, gives

(A) $m = 0.99$ for enveloped viruses

(B) $m = 1.05$ for non-enveloped viruses

(C) $m = 1.14$ for icosahedral viruses

(D) $m = 1.09$ for non-icosahedral viruses.

All logs are to the base 10. Data and formulas are presented in the Table S1.

Packaging Density	Phages	NCLDVs
Median	0.503	0.097
SD	0.458	0.164

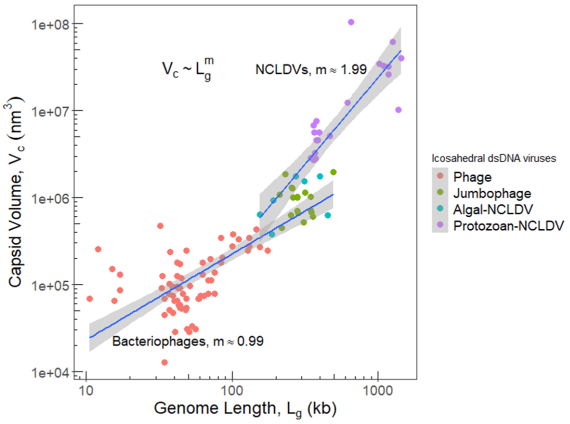

Packaging Density	Phages	NCLDVs
Median	0.454	0.102
SD	0.424	0.131

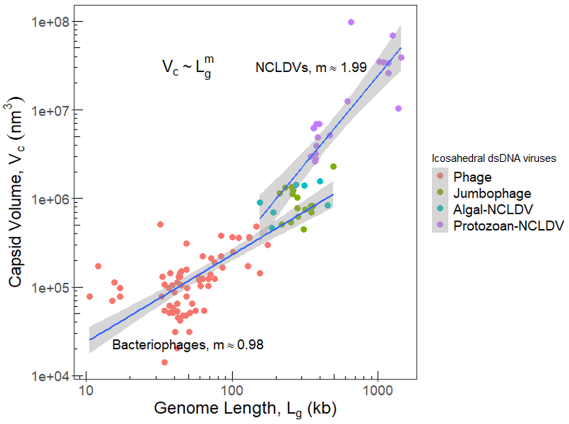

Packaging Density	Phages	NCLDVs
Median	0.461	0.102
SD	0.448	0.129

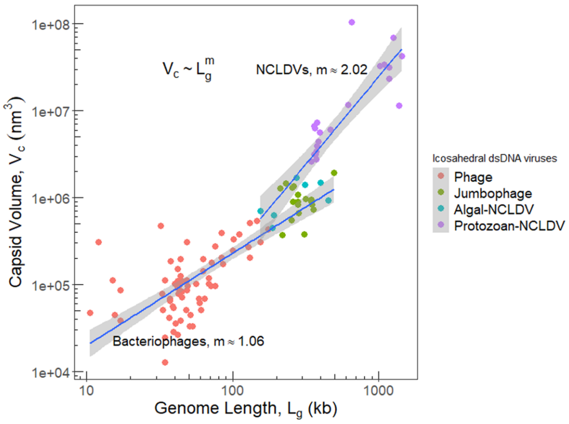

Packaging Density	Phages	NCLDVs
Median	0.496	0.097
SD	0.368	0.167

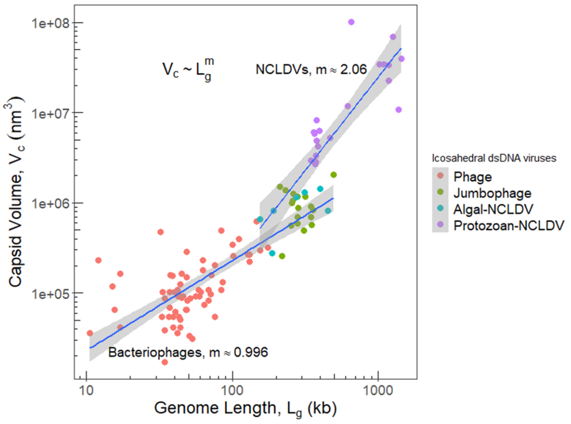

Figure S3. The log-log plot of inner capsid volume versus genome length for icosahedral dsDNA bacteriophages (including jumbophages) and NCLDVs (algal and protozoan). Related to STAR Methods "Data collection".

Inner radius estimated by subtracting capsid thickness, 3 nm for bacteriophages and 10 nm for NCLDVs, from the outer radius. The influence on the scaling relations and median packaging density of error ΔD in the capsid diameter that is randomly sampled from $[-10\text{ nm}, +10\text{ nm}]$ is shown for four separate instances. A small error $\Delta R = \Delta D/2$ in the measurement/reporting of capsid size R could give a larger error in packing density for small R as in the equation below: $\rho_{\text{exact}} = \frac{L_g}{4/3\pi R^3(1+\Delta R/R)^3} \approx \frac{L_g}{4/3\pi R^3}(1-3\Delta R/R) = \rho_{\text{calculated}}(1-3\Delta R/R)$. As can be seen from this simple calculation, the error in capsid measurement ΔR has more severe implications on density measurement for smaller R.

Table S2. Power law fit, $y = ax^m$, for capsid volume (*y*) and genome length (*x*) for group of viruses classified according to classification based on

(I) Baltimore classification

(II) Enveloped and non-enveloped

(III) Icosahedral and non-icosahedral viruses.

Related to Figure1 and STAR Methods "Data analysis".

Allometric exponents m and the associated statistical parameters (R^2 and p) for linear regression of the form $Y = mX + A$, where $Y = log\, y$ and $X = log\, x$, and $A = log\, a$ to the data for different hosts. All logs are to the base 10.

Classification	No of viruses	*m*	*A*	R^2	p-value
Baltimore classification					
dsDNA	197	1.29	3.23	0.66	$< 2.2×10^{-16}$
ssDNA	30	1.05	3.35	0.43	$5.033×10^{-05}$
dsRNA	26	0.87	3.97	0.39	0.0004
ssRNA (+)	88	1.75	2.96	0.42	$7.236×10^{-12}$
ssRNA (-)	26	3.82	1.62	0.56	$6.561×10^{-06}$
Reverse transcribing	14	1.67	3.73	0.38	0.01122
Enveloped and non-enveloped viruses					
Enveloped	113	0.99	4.40	0.63	$< 2.2×10^{-16}$
Non-enveloped	268	1.05	3.53	0.76	$< 2.2×10^{-16}$
Icosahedral and non-icosahedral viruses					
Icosahedral	291	1.14	3.55	0.69	$< 2.2×10^{-16}$
Non-icosahedral	90	1.09	3.99	0.64	$< 2.2×10^{-16}$
