# Supplementary material for: Scaling relation between genome length and particle size of viruses provides insights into viral life history
Source: iScience. 2021 Apr 21;24(5):102452. doi: 10.1016/j.isci.2021.102452 (PMC8169800; doi:10.1016/j.isci.2021.102452)
Supplement: Document S2. Article plus supplemental information [file mmc3.pdf]

iScience

CellPress
OPEN ACCESS

Article

Scaling relation between genome length and particle size of viruses provides insights into viral life history

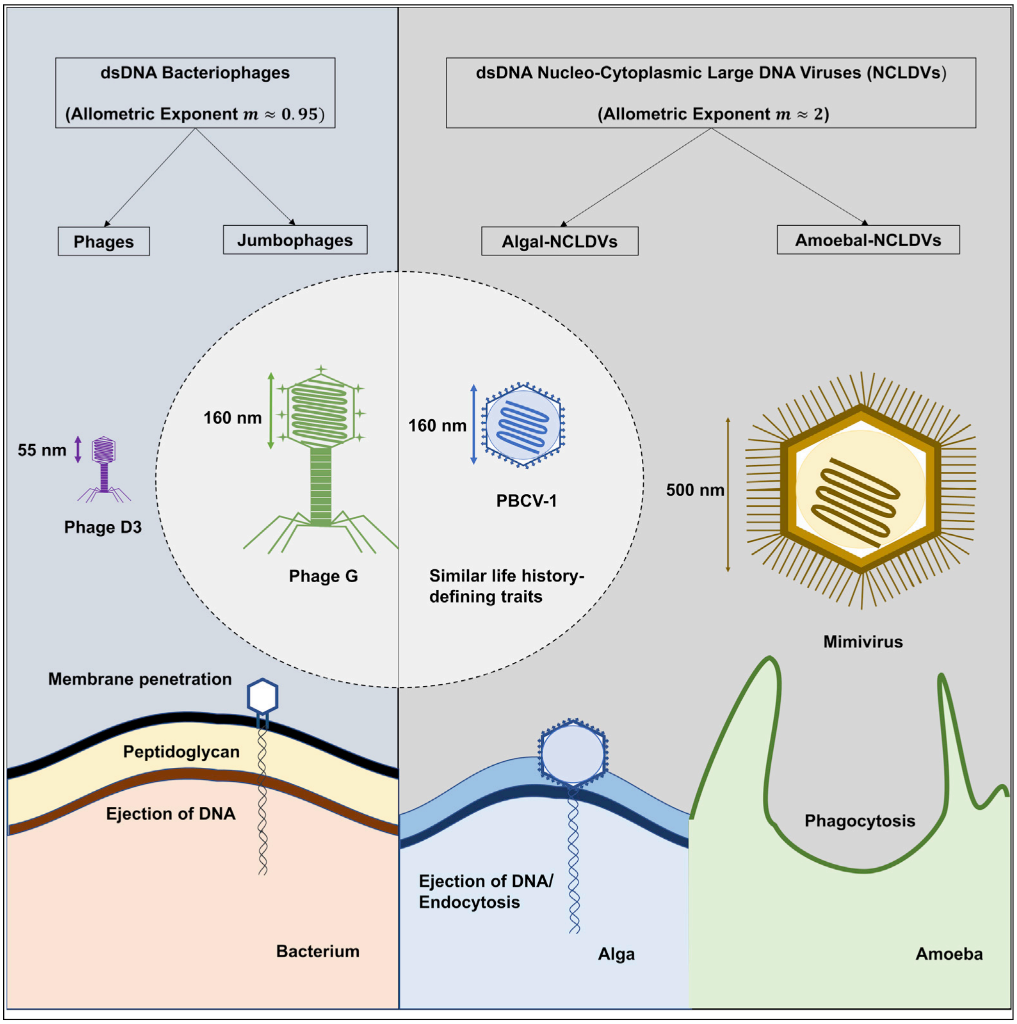

Harshali V. Chaudhari, Mandar M. Inamdar, Kiran Kondabagil

minamdar@iitb.ac.in (M.M.I.)
kirankondabagil@iitb.ac.in (K.K.)

Highlights

Capsid and genome size allometric exponent gives insights into viral life history

The allometric exponent of NCLDVs is almost twice that of bacteriophages

The exponent is largely shaped by the viral entry mechanism and capsid stability

The relaxed genome size constraint allows large viruses to evolve greater autonomy

Chaudhari et al., iScience *24*, 102452
May 21, 2021 © 2021 The Author(s).
https://doi.org/10.1016/j.isci.2021.102452

iScience

CellPress OPEN ACCESS

Article

Scaling relation between genome length and particle size of viruses provides insights into viral life history

Harshali V. Chaudhari,[1] Mandar M. Inamdar,[2,*] and Kiran Kondabagil[1,*]

SUMMARY

In terms of genome and particle sizes, viruses exhibit great diversity. With the discovery of several nucleocytoplasmic large DNA viruses (NCLDVs) and jumbo phages, the relationship between particle and genome sizes has emerged as an important criterion for understanding virus evolution. We use allometric scaling of capsid volume with the genome length of different groups of viruses to shed light on its relationship with virus life history. The allometric exponents for icosahedral dsDNA bacteriophages and NCDLVs were found to be 1 and 2, respectively, indicating that with increasing capsid size DNA packaging density remains the same in bacteriophages but decreases for NCLDVs. We argue that the exponents are largely shaped by their entry mechanism and capsid mechanical stability. We further show that these allometric size parameters are also intricately linked to the relative energy costs of translation and replication in viruses and can have further implications on viral life history.

INTRODUCTION

Viruses are obligate intracellular parasites that infect most cellular organisms in the biosphere. The simplicity of their makeup allows them to adapt and evolve rapidly to infect different life forms. They lie in a disconcerting line between the living and the nonliving. A convincing argument on the origin and diversification of viruses remains elusive. Owing to the lack of features that are key to life, such as energy generation, viruses have not found a place in the universal tree of life (Woese et al., 1990). But with the discovery of several new lineages of large complex viruses and the availability of viral genomic information, this paradigm is being reconsidered (Boyer et al., 2010; Koonin and Starokadomskyy, 2016; Forterre, 2017).

The life history of viruses includes traits, such as mode of transmission, replication rate, virus size, and burst size, that directly influence survival and reproduction (De Paepe and Taddei, 2006; Goldhill and Turner, 2014). Viruses exhibit great diversity in the type and size of their genetic material as well as the size and shape of the virus particle. The genetic material can be either single or double-stranded DNA or RNA. While the smallest virus has a genome of only 1.8 kb (single-stranded DNA genome of *Circoviruses*) (Finsterbusch and Mankertz, 2009), the largest virus (*Pandoravirus*) carries a 2.5-Mb double-stranded DNA genome (Philippe et al., 2013). Similarly, particle size can vary by up to four orders of magnitude (King et al., 2011; Cui et al., 2014). The study of size as an independent variable is a neglected area in virology. The recent discoveries of giant viruses have established size as an important ecological parameter that can have an influence on the life history of some viruses (Edwards et al., 2021). *Mimivirus* is a prototypical member of these large dsDNA viruses (Raoult et al., 2004). In terms of both genome and capsid size (1.2 Mb and ~500 nm, respectively), *Mimivirus* is bigger than several bacteria. In the last 15 years, hundreds of such giant viruses (viruses with genome size >200 kb) have been discovered, and the list is rapidly growing. Furthermore, metagenomics studies have shown that giant viruses are widespread in the environment, and their abundance in oceans is next only to that of bacterial viruses (Hingamp et al., 2013).

Giant viruses are part of a diverse group of monophyletic viruses known as nucleocytoplasmic large DNA viruses (NCLDVs) (Iyer et al., 2001). The NCLDV order belongs to the newly established phylum Nucleocytoviricota (Koonin et al., 2020; Schoch et al., 2020), and it includes seven families (Poxviridae, Asfarviridae, Iridoviridae, Ascoviridae, Phycodnaviridae, Mimiviridae, and Marseilleviridae) of viruses infecting a wide range of eukaryotes, from higher mammals to unicellular protozoans (Colson et al., 2013). Other

[1]Department of Biosciences and Bioengineering, Indian Institute of Technology Bombay, Powai, Mumbai 400076, India

[2]Department of Civil Engineering, Indian Institute of Technology Bombay, Powai, Mumbai 400076, India

*Correspondence: minamdar@iitb.ac.in (M.M.I.), kirankondabagil@iitb.ac.in (K.K.)

https://doi.org/10.1016/j.isci.2021.102452

© 2021 The Author(s).
This is an open access article under the CC BY license (http://creativecommons.org/licenses/by/4.0/).

unclassified giant viruses considered in the study are *Mollivirus* (Legendre et al., 2015; Christo-Foroux et al., 2020), *Pandoravirus* (Philippe et al., 2013; Legendre et al., 2018), *Pithovirus* (Legendre et al., 2014), *Cedratvirus* (Andreani et al., 2016; Bertelli et al., 2017), *Orpheovirus* (Andreani et al., 2018), *Faustovirus* (Reteno et al., 2015; Benamar et al., 2016), *Pacmanvirus* (Andreani et al., 2017), *Mininucleovirus* (Subramaniam et al., 2020), and *Medusavirus* (Yoshikawa et al., 2019). Most NCLDVs are icosahedral viruses, while some such as *Pandoravirus*, *Cederatvirus*, *Pithovirus,* and *Orpheovirus* are oval-shaped.

While most bacterial viruses carry genomes smaller than NCLDVs, some phages, known as jumbo phages, possess genomes of >200 kb (Yuan and Gao, 2017). Bacteriophage G, which infects *Bacillus megaterium*, is the first jumbo phage isolated and sequenced (genome size 498 kb, diameter 160 nm [Donelli, 1968; Donelli et al., 1975; Hua et al., 2017]). Other phages infecting *B. megaterium* have genome size in the range of 40–170 kb (Mihara et al., 2016). The burst size of jumbo phages ranges from 5 to 30 (Sharma et al., 2019), which is significantly less compared with other classical bacteriophages such as lambda and T4. Because viral burst size is a key fitness parameter, the smaller burst size of jumbo phages suggests higher viability of their progeny. Furthermore, comparative genomics showed that jumbo phages are phylogenetically different from the other phages, indicating their parallel evolution (Hua et al., 2017; Yuan and Gao, 2017)

Thus, there appears to be a greater degree of genome and particle size variation in both prokaryotic and eukaryotic viruses. While hosts are known to influence viral physiology, factors that play important roles in shaping these two important viral parameters, namely, particle size and genome size, have not been understood. In this study, we investigate how the size of viruses, especially large size (of both particle and genome), manifests in relation to their life history within its host. We also endeavor to gain insights into the possible evolutionary pressures exerted on the viruses by the competing organisms in the environment and the propellants of size diversity across NCLDV families. Although such an analysis based on two variables is coarse-grained, it serves to fill important gaps in understanding the characteristics influencing the life history of viruses.

RESULTS AND DISCUSSION

The relationship among all viruses (capsid volume, genome size, gene number) and their hosts

Viruses exhibited a wide range of distribution with both genome size and capsid volume (Figure 1). If all the viruses are considered, irrespective of the host, there appears to be a linear relationship between the external capsid volume and genome length with an exponent of 1.13 (p value $< 2.2 \times 10^{-16}$, $R^2 = 0.67$) when plotted on a log-log scale of base 10. There is no clear demarcation of either capsid or genome size range as per the host as each host can get infected by viruses of different sizes. An exponent of more than one suggests an out-of-proportion increase in the capsid volume compared with the genome length that implies a lesser packaging density for viruses with bigger capsids. A previous study determined the exponent with 88 viruses to be around 1.4 without considering the identity of the host (Cui et al., 2014), drawing a conclusion that prediction of virion size is possible from genome length using a simple scaling law. Here, we reexamine the relationship with a larger data set of 381 viruses (Table S1) and update the exponent (see Figures S1 and S2, and Table S2).

Genome size and capsid volume do not appear to scale proportionally in the case of RNA viruses when compared with the DNA viruses (Figure 1). Capsid volume variation of RNA viruses is about three orders of magnitude, while their genome size varies by about an order of magnitude. This variation in the physical parameters appears to be intricately linked to the life history traits of viruses (multiplication rate, viability, and mode of transmission/entry into host cell) that are ultimately related to their life cycle. In most RNA viruses, the inherent error-prone replication without proof-reading imposes a constraint on the genome size (Wellehan et al., 2016). In addition, there appear to be two mechanistically different pathways for capsid assembly in RNA viruses that could influence the capsid size (Perlmutter et al., 2014; Chevreuil et al., 2018; Valbuena et al., 2020). While the first mechanism involves a rapid, disordered, and random binding of capsid monomers to genomic RNA followed by rearrangements and ordering of capsid, the second pathway starts with the nucleation of capsid protein around the genomic RNA and its sequential growth leading to the completion of the assembly. In both mechanisms, the secondary and tertiary structure of the RNA genome and the preferred curvature of the assembled capsid emanating from the specific protein-protein interactions appear to influence the capsid size (Perlmutter et al., 2013; Beren et al., 2017). Thus, the dependence of the capsid size on the secondary and tertiary structure of the genomic RNA, along with its genome length (Hu et al., 2008), could possibly explain the wide variation of capsid volume in RNA

CellPress
OPEN ACCESS

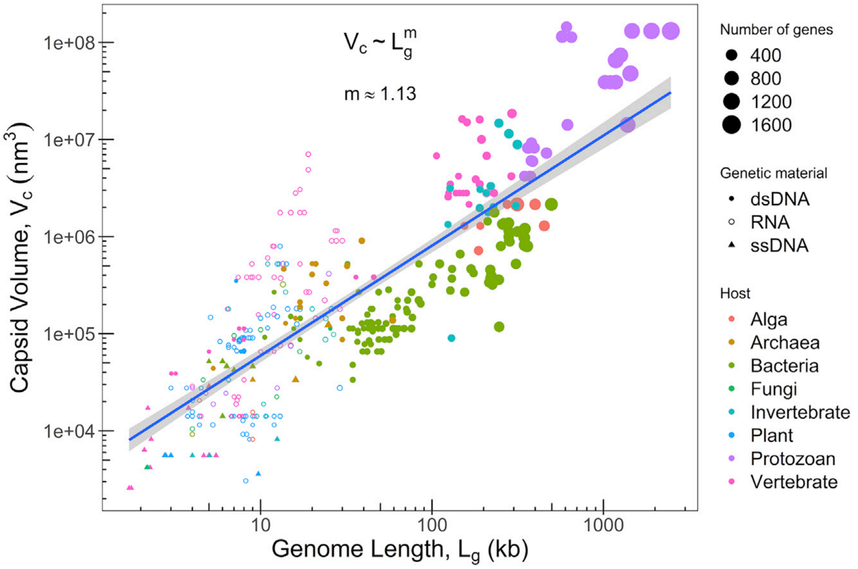

Figure 1. The log-log plot of outer capsid volume as a function of genome length for viruses infecting different hosts

Size of the data points indicate the number of genes while shapes are in accordance with the genetic material (filled circle-dsDNA viruses, empty circle-RNA viruses, filled triangle-ssDNA viruses). A power law $y = ax^m$ is used as a fitting expression for the entire data and appears as a straight line on the log-log plot. A linear regression fit of the form $Y = mX + A$ to the data, where $Y = \log y$, $X = \log x$, and $A = \log a$, gives $A = 3.64$ and $m = 1.13$ (p value $< 2.2 \times 10^{-16}$ and $R^2 = 0.67$). All logs are to the base 10. Formulas to calculate capsid volume are described in STAR methods and data are available in the Table S1. See also Figure S1 and S2 and Table S2.

viruses (Figure 1). While the largest RNA virus has a genome size of 33.5 kb (Nidovirus), the average size appears to be about 10 kb (Saberi et al., 2018). In the case of RNA viruses, the exponent was 1.95 but the R^2 value was low (0.39). The packaging densities of RNA viruses were not estimated as the genome volume could not be calculated with confidence.

About 197 of 381 (50%) viruses we considered are dsDNA viruses. Table 1 summarizes the hosts and exponents calculated for the group of dsDNA viruses. The p and R^2 values of archaea and invertebrate subgroups are not significant, which might be owing to the small sample size, making it difficult to comment on the relationship between particle size and genome size among these subgroups. Viruses infecting vertebrate and unicellular eukaryotes such as protozoan and algae exhibit a slope of greater than 1 with a p value of 1.07×10^{-13} and R^2 value of 0.87, while interestingly, bacteria infecting viruses exhibit a slope of less than 1 with a p value $< 2.2 \times 10^{-16}$ and R^2 value of 0.66.

The dsDNA viruses showed an increase in gene numbers with increasing genome length. Particularly, giant viruses with larger genomes also exhibit coding densities similar to that of smaller viruses (Van Etten et al., 2010; Philippe et al., 2013; Bajrai et al., 2016) and hence code for a large number of genes (Figure 1). Among icosahedral dsDNA viruses, the data were subdivided into viruses infecting bacteria and viruses of the NCLDV family that mostly infect unicellular eukaryotes such as protozoan and algae. NCLDVs such as poxviruses that infect higher multicellular eukaryotes and other non-NCLDV eukaryotic dsDNA viruses such as herpesvirus, adenovirus, and so on were excluded from this study considering their wide host range (which led to different life histories) and host cell size. Bacteriophages are the most studied group of viruses, facing different environments and evolutionary pressures to those faced by NCLDVs that infect protozoan or alga. Hence, a comparative study of these two subsets will serve as a base to understand the evolution of these viruses.

The allometric relationship between genome length and particle size of icosahedral dsDNA bacteriophages and NCLDVs

The regression gives an exponent of about 0.95 for the bacteriophage subgroup with an R^2 value of 0.70 and a significant p value ($<2.2 \times 10^{-16}$). The exponent from the regression line of NCLDVs is 2.00, with

Table 1. Power law fit, $y = ax^m$, for capsid volume (y) and genome length (x) for each group of dsDNA viruses classified as per their host

Host	No of dsDNA viruses	*m*	*A*	R^2	p value
Vertebrates	29	1.25	3.88	0.87	1.07×10^{-13}
Invertebrates	13	2.49	0.67	0.34	0.02
Bacteria	106	0.87	3.70	0.66	$<2.2 \times 10^{-16}$
Archaea	15	0.58	4.61	0.09	0.15
Protozoans, algae	33	1.96	1.71	0.70	5.37×10^{-10}

Allometric exponents m, proportionality coefficient A and the associated statistical parameters (R^2 and p value) for linear regression of the form $Y = mX + A$, where $Y = \log y$ and $X = \log x$ and $A = \log a$ to the data for different hosts. All logs are to the base 10. See also Figure S1A.

an R^2 value of 0.73 and a p value of 4.87×10^{-9}. The exponents of these subgroups (0.95 and 2.00) fall on either side of the regression line exponent of viruses across different hosts (1.13) (Figure 2). The exponent values suggest that in the case of bacteriophages both the parameters are increasing in proportion with each other, while in NCLDVs, the capsid volume is increasing at a much higher magnitude than their genome length. Consequently, the genome packaging density of NCLDVs is lesser compared to the bacteriophages.

We hypothesize that the particle size and genome size intersect at two stages in the virus life cycle. The first is when a virus enters the host cell, and the second is during genome packaging and capsid assembly. The mode of entry might have a huge impact on the optimization of particle and genome sizes of viruses. Capsids less than a threshold size cannot accommodate large genomes, while smaller genomes may not create enough internal pressure inside the capsid required for genome injection, which is inevitable for successful infection by most bacteriophages (Nurmemmedov et al., 2007). The NCLDVs, on the other hand, appears to have no such prerequisite for infection, as whole virus particles enter the host cell by either phagocytosis or endocytosis, eliminating the role of viral packaging density on their infection capability.

Although dsDNA bacteriophages exhibit size variability, an overwhelming majority of them (~85%) have an icosahedral structure (Ackermann, 2003). Amoeba-infecting giant viruses, on the other hand, appear to have a far more diversity in shape, including spherical, icosahedral, ovoidal, and icosahedron with a tail. This points to a lack of evolutionary pressure on the shape of giant viruses, whereas icosahedral bacteriophages do not have the same independence to change their shape. A simulation study on semiflexible polymers showed that sphere-shaped capsids package and eject genome at a much faster rate (Ali et al., 2006). Icosahedron, being similar to a sphere where pressure is exerted upon the genome from all sides facilitating genome injection into the host, might be as per bacteriophages to maintain their infectious capacity.

Giant viruses enter the amoebal host by phagocytosis. Because ameba appears to indiscriminately engulf and phagocytose particles of size 0.5 μm or greater (Korn and Weisman, 1967; Raoult and Boyer, 2010), it appears that capsid size—rather than its shape and internal capsid pressure (see the next section)—is critical for infection. Because ameba harbors a gamut of microbes such as bacteria, viruses, algae, and fungi, the larger particle size of giant viruses might have resulted from the competition with microorganisms to be phagocytosed (Slimani et al., 2013). Furthermore, genetic recombination events with other microbial genomes in the amoebal "melting pot" could have led to the size expansion of giant viral genomes (Boyer et al., 2009; Colson and Raoult, 2010). Giant viruses have been shown to accumulate genes that encode repeat-domaining proteins (Shukla et al., 2018), transpovirions, and provirophages in high copy numbers (Desnues et al., 2012). While the larger of the giant viruses such as *Mimivirus*, *Pithovirus*, *Pandoravirus*, and so on enter the host cell by phagocytosis, the smaller *Marseillevirus* that has a capsid of only about 250 nm in diameter has evolved to enter via pinocytosis or by phagocytosis of aggregates of several viral particles (Arantes et al., 2016). Such variation in the entry mechanism is not observed in icosahedral bacteriophages.

Packaging density of dsDNA icosahedral bacteriophages and NCLDVs

Although the scaling exponents provide us great insights into how the capsid size evolves with genome size, they do not directly inform us about how tightly the DNA is packaged within the viral capsids and

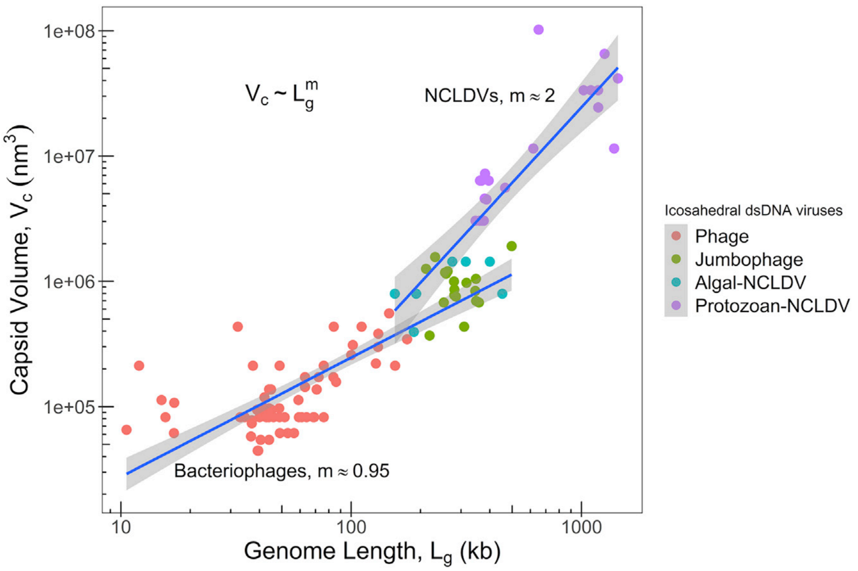

Figure 2. The log-log plot of inner capsid volume versus genome length for icosahedral dsDNA bacteriophages (including jumbo phages) and NCLDVs (algal and protozoan)

Inner radius estimated by subtracting capsid thickness, 3 nm for bacteriophages and 10 nm for NCLDVs, from the outer radius. A power law $y = ax^m$ appears as a straight line on the log-log plot and is used to independently fit the data for bacteriophages and NCLDVs. For that linear regression fit of the form $Y = mX + A$ is performed over the data, where $Y = \log y$, $X = \log x$, and $A = \log a$. Parameters for phage are $A = 3.49$, $m = 0.95$ (p value $< 2.2 \times 10^{-16}$ and $R^2 = 0.70$) and for NCLDVs are $A = 1.38$, $m = 2.00$ (p value $= 4.87 \times 10^{-9}$.and $R^2 = 0.73$). All logs are to the base 10. Data and formulas are presented in the Table S1.

its possible connection with the viral infection mechanism. It is well-known from the literature that the so-called pressure within the viral capsids is related to the spontaneous ejection of DNA from the viral capsid during infection (Evilevitch et al., 2003; Tzlil et al., 2003). Bacteriophages infect the host by injecting their DNA into the host, while the capsid stays outside. On the other hand, NCLDVs bring about host infection by phagocytosis in which the entire virus is ingested into the host. Hence, it is reasonable to expect that the internal pressure inside the phage capsid is generally higher than that within NCLDVs. Because it is known from the literature that the pressure within the capsid increases with packaging density (Rau et al., 1984; Purohit et al., 2005; Bauer et al., 2015), we hypothesize that statistically speaking, the packaging density within bacteriophages should be greater than that in the NCLDVs. Indeed, from our data (see STAR methods and Figure S3), we find that the median value of the DNA packing fraction within the dsDNA icosahedral phages is approximately 0.47 (standard deviation – 0.23) as compared with the much lower value of 0.09 (standard deviation – 0.14) for dsDNA icosahedral/spherical NCLDVs. Thus, our study gives us another potential connection between viral allometry (packing density) and life history (infection mechanism).

The "overlapping region"

Another unexpected finding from our studies comes from exploring the "overlapping region" between bacteriophages and NCLDVs in Figure 2. Although on average, the allometric relation between the bacteriophages and NCLDVs is quite different, owing to statistical fluctuations a few data points from smaller NCLDVs and larger bacteriophages (or jumbo phages) seem to be mixed in this region. This prompted us to ask a very naive question: given this overlap, are there also any similarities between jumbo phages and smaller NCLDVs? Surprisingly, we found that jumbo phages have an ancestry that is significantly branched off from both smaller bacteriophages and NCLDVs (Yuan and Gao, 2017). Interestingly, jumbo phages have a few important life-history traits that are remarkably similar to those of NCLDVs. For example, jumbo phages, unlike smaller bacteriophages, are believed to maintain viral factory-like structures during their life cycle (Chaikeeratisak et al., 2017). Compartmentalization helps bigger viruses to protect their genome from antivirals and nucleases as well as replication and transcription processes take place efficiently by concentrating required factors. Moreover, similar to NCLDVs, jumbo phages have more genes

associated with genome replication and nucleotide metabolism, as compared with smaller phages (Yuan and Gao, 2017). On the other front, smaller NCLDVs, which are typically algae-infecting viruses, have life-history traits that also show certain similarities with bacteriophages. For example, during infection, Paramecium bursaria chlorella virus (a member of the Phycodnaviridae family that infects the unicellular green alga *Chlorella* sp.), such as bacteriophages, only inject its DNA into the host while leaving the capsids outside (Van Etten, 2003). Similarly, some of these viruses also exhibit pseudolysogeny (Van Etten et al., 1991; Thomas et al., 2011), which is a common phenomenon observed in bacteriophages that helps in the long-term survival of viruses in unfavorable environments (Abedon, 2008). These observations suggest that though the phylogeny of bacteriophages and NCLDVs are divergent, virus size in itself could influence the life-history traits of viruses.

Potential implications of values of m in the relation $V_c \sim L_g^m$ for viral life history

From Figure 2, we obtained that the viral capsid volume V_c is connected to the packaged genome length L_g

$$V_c \sim L_g^m, \qquad \text{(Equation 4)}$$

where the exponent $m \approx 1$ for phages and $m \approx 2$ for NCLDVs. Because $V_c \sim r_c^3$, where r_c is the capsid inner radius, Equation 4 can be re-written as

$$r_c \sim L_g^{m/3}. \qquad \text{(Equation 5)}$$

The difference in the m values of phages and NCLDVs leads to interesting implications as discussed in the following text.

Implication of m for viral capsid stability

As discussed earlier in the section on DNA packaging density, the pressure in the viral capsid is generally an increasing function of DNA packaging density (Rau et al., 1984; Purohit et al., 2005; Bauer et al., 2015). Hence, the effective internal pressure in the capsid is as follows:

$$p_c(\rho) = p_c\left(L_g / r_c^3\right), \qquad \text{(Equation 6)}$$

where, p_c is an increasing function of its argument. We use the same symbol p_c in both sides of Equation 6 for notational economy. Using Equations 5 in 6, we get that $p_c(\rho) = p_c\left(r_c^{\frac{3}{m}-3}\right)$. It is known that tangential tension is generated in the walls of spherical pressure vessels (also called as hoop stress) due to internal pressure (Barber, 2010). By modeling the virus as one such with internal pressure p_c, we can obtain the tensile stress in the viral capsid walls as $\sigma = p_c r_c / t = p_c\left(r_c^{\frac{3}{m}-3}\right) r_c / t$ using simple mechanical considerations, where t is the thickness of the capsid and $t \ll r_c$ (thin walls) (Barber, 2010). For the capsid to remain intact and not burst, the tensile stress σ should be less than the ultimate tensile strength σ_u of the capsid walls. Hence,

$$\sigma_u > \sigma = \frac{p_c\left(r_c^{\frac{3}{m}-3}\right) r_c}{t}. \qquad \text{(Equation 7)}$$

We see from this simple expression that the tensile stress increases with size for the same amount of internal pressure.

During capsid self-assembly, high mechanical stresses could be generated in the capsid surface owing to a combination of growth and Gaussian curvature. This stress could be relieved by creating topological defects in the icosahedral capsid surface or by inducing anisotropy in its growth and creating elongated capsids (Castelnovo, 2017). However, it is known from theoretical considerations, numerical simulations, and experiments that viral capsids still retain residual stress (Klug et al., 2012; Zandi et al., 2020). In our simple calculation, we take the residual stress in the capsid as a given, and the pressure created by the packaged DNA simply adds tensile stress (Equation 7) to the capsid. It was shown in a numerical study that for viral capsids with higher triangulation number T, the residual tension at the hexamer locations is high, though the increase in tension with T is not always monotonic (Zandi and Reguera, 2005). Similarly, it is also observed that the size of individual capsomeres is very similar for different viruses. In that case, $T \sim r_c^{\alpha}$,

where $\alpha \approx 0.5$, that is, capsid T number increases with increasing capsid radius (Božič et al., 2013). Thus, the residual stress in the capsid at some locations should generally increase with the capsid size. The study of intricate details of force generation in the capsid owing to a combination of DNA pressure and capsid self-assembly residual stresses is beyond the scope of this article. However, the residual tension could reduce the effective ultimate tensile strength of the capsid walls $\sigma_u(r_c)$ (Zandi and Reguera, 2005), which, from the aforementioned discussion, is expected to decrease with r_c. Because $m \approx 1$ in the case of bacteriophages, for capsid integrity $\sigma_u(r_c) > \frac{p_c(1) r_c}{t}$, where the term $p_c(1)$ indicates that the capsid pressure varies little with phage size. Hence, as the capsid size increases, the capsid wall stress increases with r_c, while its ultimate tensile strength $\sigma_u(r_c)$ decreases, making the capsid failure-prone. Indeed, jumbo phages have decoration proteins that are known to provide structural strength to the capsid (Effantin et al., 2013). However, the production of decoration proteins should require additional resources from the host and increased information in the viral genome.

For NCLDVs, r_c increases to sizes that are up to three times the size of jumbo phages. As a result, for the same internal pressure p_c as for the phages, the capsid wall stress will increase many folds, on the one hand, while σ_u keeps decreasing on the other. Hence, the reinforcement requirement for sustaining capsid integrity would increase many times when compared to even jumbo phages. However, it seems that NCLDVs have evolved another strategy to counter the threat of structural failure. In their case, because $m \approx 2$, the structural integrity condition becomes $p_c({r_c}^{-3/2}) < \frac{\sigma_u}{r_c} t$. Hence, the integrity condition could be satisfied because, while the term on the right-hand side decreases with increasing capsid size, as discussed previously, the pressure $p_c(r_c^{-3/2})$ on the left-hand side, unlike for phages, also simultaneously decreases. As a result, NCLDVs could maintain their capsid integrity with respect to internal pressure owing to the genome. Thus, the scaling exponent $m \approx 2$ in $V_c \sim L_g^m$ could have this important implication for NCLDVs.

Implication of allometric exponent *m* for metabolic cost of viral life history

DNA replication and translation of capsid proteins are two of the most energy intense events in viral life history (Mahmoudabadi et al., 2017). As discussed in the previous subsection, assuming that the capsomere sizes remain approximately constant across different viruses, the number of capsid proteins $N_c \sim {r_c}^2$, the approximate area of the capsid surface (Mahmoudabadi et al., 2017). Hence, the energy cost of translating capsid protein(s) for a single virus (Mahmoudabadi et al., 2017) scales as

$$E_{tl} \sim r_c^2. \qquad \text{(Equation 8)}$$

Similarly, because the energy cost of replicating DNA of a single virus $E_{rep} \sim L_g$ (Mahmoudabadi et al., 2017), using Equation 5, we get that

$$E_{rep} \sim r_c^{3/m} \qquad \text{(Equation 9)}$$

As seen before, $m \approx 1$ for bacteriophages, owing to which $E_{rep} \sim r_c^3$. Hence, when r_c is small, the translation cost $E_{tl} \sim r_c^2$ is expected to dominate over the replication cost $E_{rep} \sim r_c^3$. The reverse should be true when the size r_c of virus increases (Mahmoudabadi et al., 2017), especially for NCLDVs as they have large capsid sizes compared with most bacteriophages. Hence, if the same $m \approx 1$ for bacteriophages were applicable for NCLDVs, we would expect E_{rep} to dominate over E_{tl} for these viruses. However, as seen earlier, for NCLDVs $m \approx 2$, owing to which $E_{rep} \sim r_c^{3/2}$, and the translation cost $E_{tl} \sim r_c^2$ should dominate over the replication cost for NCLDVs even for larger capsid sizes. The replication cost for NCLDVs in terms of r_c can be obtained by using the scaling relation expressed in Equations 4 in 1. This scaling relation could be written more explicitly as $V_c = \frac{4}{3}\pi r_c^3 = aL_g^m$, from which $L_g = \left(\frac{4\pi}{3a}\right)^{1/m} r_c^{3/m}$. The exponent $m = 2.00$ and the proportionality coefficient $a = 10^{1.38}$ were obtained for NCLDVs using the fitting procedure described earlier (see Figure 2 caption, STAR methods section). As expected, this relation well represents the energetic cost E_{rep} as a function of capsid radius for NCLDVs (Figure 3).

What could be the potential implication of this finding for viral life history? According to Mahmoudabadi et al. (2017) and our arguments presented previously, the cost associated with making capsid proteins dominate the energy budget. The fact that protozoan and algal giant viruses exist in large numbers (Monier et al., 2008; Hingamp et al., 2013; Li et al., 2019) suggests that a bigger size is beneficial to viruses in certain host/environmental niches. Furthermore, the finding that many NCLDVs have independently evolved bigger sizes (Filee, 2013) suggests common evolutionary mechanisms of size expansion that are yet to be

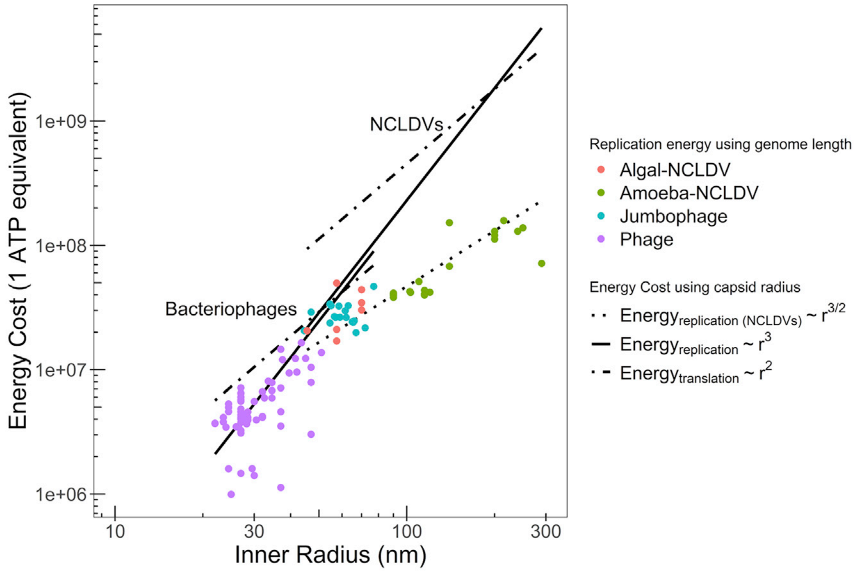

Figure 3. The log-log plot of translation and replication energy cost of icosahedral dsDNA bacteriophages and NCLDVs as a function of their inner radius

The replication cost is obtained using genome length (Equation 1) and capsid radius (Equation 2). The translation cost for capsid protein molecules is obtained from Equation 3. The gap in the energy costs of NCLDVs and bacteriophages is because of the difference in the thickness *t* of their viral capsids (Equation 3). Energy costs are reported in terms of the number of ATP hydrolysis events (Mahmoudabadi et al., 2017). As discussed in Mahmoudabadi et al. (2017) translation ($\sim r_c^2$) and replication ($\sim r_c^3$) rates dominate at lower and higher capsid sizes, respectively. This trend works well for bacteriophages (data points for replication cost using actual length). For NCLDVs, however, the translation cost always dominates because $L_g \sim r_c^{3/2}$. See text for more detail. All logs are to the base 10. The entire data are represented in the Table S1.

understood. If the energy budget of the host is indeed a limiting factor in the case of bigger viruses, the number of progenies will have to be sacrificed. In this case, however, if the fitness of each virion increases, say, owing to an increase in virion stability because of low DNA packaging, as argued earlier (Edwards et al., 2021), then only a fewer of them are needed for continued sustenance. The larger size of the virion is also helpful for phagocytosis (Rodrigues et al., 2016). If the energy budget of the host is not a limiting factor, then all these considerations are still relevant but without the strict constraint on the progeny number.

What is the advantage of such big capsids for evolution? A larger capsid size can, in principle, help viruses accrue more genetic content. Because we have argued that the cost of DNA replication is a small fraction of the energy required for producing capsid protein(s), the cost burden of this acquisition is minimal. If, in addition, it does not affect the overall fitness of the virus, the newly acquired DNA can explore the evolutionary landscape via genetic drift. It was recently proposed that in the case of *Pandoravirus* (Legendre et al., 2019), some acquired noncoding DNA have transitioned to protein-coding ones *de novo*. Furthermore, the newborn genes were found to be under "slight" negative selection pressure indicating that they are in the process of fixation (Legendre et al., 2019). Thus, it appears that capsid expansion confers higher evolvability to some giant viruses in certain host niches leading to their diversification. In the process, giant viruses contribute novel genes to their environment via mobilomes in the protozoan "melting pot" (Desnues et al., 2012; Colson et al., 2017).

The two parameters of the virus, capsid radius R_c and genome size L_g have important implications for the life history of viruses. By increasing L_g, the virus can have greater complexity because its ability to translate more proteins increases. On the other hand, capsid volume $V_c \sim r_c^3$ provides a cap on how big their genome could be. Having a large packaging density $\rho \sim L_g/r_c^3$, loosely speaking, leads to higher internal pressure within the capsid which is widely believed to be used by bacteriophages to inject their DNA into the host cells during infection (Grayson et al., 2006; Brandariz-Nuñez et al., 2019). However, large internal pressure combined with large capsid size could make the virus susceptible to mechanical failure by tearing of the capsid wall owing to tension. Hence, many large phages have decorating proteins and/or cementing proteins to safeguard against rupture. Manufacturing of these additional ingredients, nevertheless, require energy and

material resources from the host. The really large NLCDVs, however, are even more susceptible to capsid rupture. But as discussed previously, the fact that the DNA packaging density decreases with increasing size of NCLDVs could help reduce the internal pressure for larger viruses. Moreover, NCLDVs do not require high internal pressure to inject their genome into their protozoa host because they carry out the infection by entering into the host by phagocytosis. In fact, the large size of NCLDVs is comparable with that of many bacteria that are also ingested for nutrition by protozoa via phagocytosis. Hence, it is conceivable that the NCLDVs are well placed in terms of size for ingestion into their host.

Conclusion

In this study, by meticulously analyzing the capsid and genome sizes of a large number of viruses, we show that the allometric exponent between these two parameters gives us insights into the evolution and life histories of viruses. A major finding from this study is that the allometric exponent of NCLDVs is almost twice that of bacteriophages. In the case of bacteriophages, the need to maintain the internal capsid pressure, which is essential for infection, seems to impose a major constraint on genome evolution. On the other hand, the constraint on genome evolution seems to be relaxed in the case of many unicellular eukaryotes-infecting NCLDVs that have evolved larger capsids for a lesser amount of DNA (and hence exhibit a higher exponent) to suit their mode of infection while maintaining capsid stability. Consequently, larger capsids could potentially accommodate additional DNA without adversely affecting its energy budget. We propose that this feature has helped in the evolution of larger viruses with greater autonomy. Overall, we suggest plausible reasons for the interplay between genome and particle sizes as important life-history determinants of viruses.

Limitation of the study

While we have taken utmost care in gathering our data, there could be some variations in the capsid sizes (e.g. owing to pH conditions) and capsid thickness than the ones used in the article. These differences could produce some errors in the calculated DNA packaging density, especially for smaller capsids. Our findings regarding the allometric exponent and the associated implications are applicable in a statistical sense, and there are viruses within the group that do not follow the expected trend. Based on the literature, we take DNA replication and capsid protein production to have the highest energy costs for the virus. However, the energy cost for making other gene products could also be high, especially for NCLDVs. We implicate the tension in capsid walls owing to DNA packaging pressure to be a determinant of capsid mechanical stability. However, we could not account for environmental conditions, such as temperature and pH, that could modify interactions between capsomeres and be significant for capsid stability.

STAR★METHODS

Detailed methods are provided in the online version of this paper and include the following:

- KEY RESOURCES TABLE
- RESOURCE AVAILABILITY
  - Lead contact
  - Materials availability
  - Data and code availability
- METHOD DETAILS
  - Data collection
- QUANTIFICATION AND STATISTICAL ANALYSIS
  - Volume and packaging density calculation
  - Data analysis
  - Calculation of viral energetic cost
  - Replication energy
- TRANSLATION ENERGY

SUPPLEMENTAL INFORMATION

Supplemental information can be found online at https://doi.org/10.1016/j.isci.2021.102452.

ACKNOWLEDGMENTS

K.K. acknowledge the support of Department of Science and Technology, SERB [EMR/2016/005155], the Department of Biotechnology, DBT [BT/PR35928/BRB/10/1841/2019] and The Board of Research in

Nuclear Sciences, BRNS [58/14/11/2020-BRNS/37188] grants. M.M.I and K.K. acknowledge the support of Science and Engineering Research Board, SERB [MSC/2020/000536]. H.V.C. acknowledges the support of DST-INSPIRE Senior Research Fellowship. M.M.I. acknowledges helpful exchanges with R. Phillips and G. Mahmoudabadi. We thank the anonymous reviewers for their helpful comments.

AUTHOR CONTRIBUTIONS

H.V.C., M.M.I., and K.K. conceived and designed the research. H.V.C. performed the research. All the authors analyzed the data and wrote the manuscript.

DECLARATION OF INTERESTS

The authors declare no competing interests.

Received: January 18, 2021
Revised: March 23, 2021
Accepted: April 15, 2021
Published: May 21, 2021

REFERENCES

Abedon, S.T. (2008). Bacteriophage Ecology: Population Growth, Evolution, and Impact of Bacterial Viruses (Cambridge University Press).

Ackermann, H.-W. (2003). Bacteriophage observations and evolution. Res. Microbiol. *154*, 245–251. https://doi.org/10.1016/S0923-2508(03)00067-6.

Ali, I., Marenduzzo, D., and Yeomans, J.M. (2006). Polymer packaging and ejection in viral capsids: shape matters. Phys. Rev. Lett. *96*, 208102. https://doi.org/10.1103/PhysRevLett.96.208102.

Andreani, J., Aherfi, S., Bou Khalil, J.Y., Di Pinto, F., Bitam, I., Raoult, D., Colson, P., and La Scola, B. (2016). Cedratvirus, a double-cork structured giant virus, is a distant relative of pithoviruses. Viruses *8*, 300. https://doi.org/10.3390/v8110300.

Andreani, J., Khalil, J.Y.B., Sevvana, M., Benamar, S., Di Pinto, F., Bitam, I., Colson, P., Klose, T., Rossmann, M.G., and Raoult, D. (2017). Pacmanvirus, a new giant icosahedral virus at the crossroads between Asfarviridae and Faustoviruses. J. Virol. *91*, e00212–e00217. https://doi.org/10.1128/JVI.00212-17.

Andreani, J., Khalil, J.Y.B., Baptiste, E., Hasni, I., Michelle, C., Raoult, D., Levasseur, A., and La Scola, B. (2018). Orpheovirus IHUMI-LCC2: a new virus among the giant viruses. Front. Microbiol. *8*, 2643. https://doi.org/10.3389/fmicb.2017.02643.

Arantes, T.S., Rodrigues, R.A.L., Dos Santos Silva, L.K., Oliveira, G.P., de Souza, H.L., Khalil, J.Y.B., de Oliveira, D.B., Torres, A.A., da Silva, L.L., Colson, P., et al. (2016). The large marseillevirus explores different entry pathways by forming giant infectious vesicles. J. Virol. *90*, 5246–5255. https://doi.org/10.1128/JVI.00177-16.

Bajrai, L.H., Benamar, S., Azhar, E.I., Robert, C., Levasseur, A., Raoult, D., and La Scola, B. (2016). Kaumoebavirus, a new virus that clusters with Faustoviruses and Asfarviridae. Viruses *8*, 278. https://doi.org/10.3390/v8110278.

Barber, J.R. (2010). Intermediate Mechanics of Materials (Springer Science & Business Media). https://doi.org/10.1007/978-94-007-0295-0.

Bauer, D.W., Li, D., Huffman, J., Homa, F.L., Wilson, K., Leavitt, J.C., Casjens, S.R., Baines, J., and Evilevitch, A. (2015). Exploring the balance between DNA pressure and capsid stability in herpesviruses and phages. J. Virol. *89*, 9288–9298. https://doi.org/10.1128/JVI.01172-15.

Benamar, S., Reteno, D.G.I., Bandaly, V., Labas, N., Raoult, D., and La Scola, B. (2016). Faustoviruses: comparative genomics of new Megavirales family members. Front. Microbiol. *7*, 3. https://doi.org/10.3389/fmicb.2016.00003.

Beren, C., Dreesens, L.L., Liu, K.N., Knobler, C.M., and Gelbart, W.M. (2017). The effect of RNA secondary structure on the self-assembly of viral capsids. Biophysical J. *113*, 339–347. https://doi.org/10.1016/j.bpj.2017.06.038.

Bertelli, C., Mueller, L., Thomas, V., Pillonel, T., Jacquier, N., and Greub, G. (2017). 'Cedratvirus lausannensis–digging into Pithoviridae diversity'. Environ. Microbiol. *19*, 4022–4034. https://doi.org/10.1111/1462-2920.13813.

Boyer, M., Yutin, N., Pagnier, I., Barrassi, L., Fournous, G., Espinosa, L., Robert, C., Azza, S., Sun, S., Rossmann, M.G., et al. (2009). Giant Marseillevirus highlights the role of amoebae as a melting pot in emergence of chimeric microorganisms. Proc. Natl. Acad. Sci. U S A *106*, 21848–21853. https://doi.org/10.1073/pnas.0911354106.

Boyer, M., Madoui, M.-A., Gimenez, G., La Scola, B., and Raoult, D. (2010). Phylogenetic and phyletic studies of informational genes in genomes highlight existence of a 4 th domain of life including giant viruses. PLoS One *5*, e15530. https://doi.org/10.1371/journal.pone.0015530.

Božič, A.L., Šiber, A., and Podgornik, R. (2013). Statistical analysis of sizes and shapes of virus capsids and their resulting elastic properties. J. Biol. Phys. *39*, 215–228. https://doi.org/10.1007/s10867-013-9302-3.

Brandariz-Nuñez, A., Liu, T., Du, T., and Evilevitch, A. (2019). Pressure-driven release of viral genome into a host nucleus is a mechanism leading to herpes infection. Elife *8*, e47212. https://doi.org/10.7554/eLife.47212.

Brister, J.R., Ako-Adjei, D., Bao, Y., and Blinkova, O. (2015). NCBI viral genomes resource. Nucleic Acids Res. *43*, D571–D577. https://doi.org/10.1093/nar/gku1207.

Castelnovo, M. (2017). Viral self-assembly pathway and mechanical stress relaxation. Phys. Rev. E *95*, 52405. https://doi.org/10.1103/PhysRevE.95.052405.

Chaikeeratisak, V., Nguyen, K., Egan, M.E., Erb, M.L., Vavilina, A., and Pogliano, J. (2017). The phage nucleus and tubulin spindle are conserved among large Pseudomonas phages. Cell Rep. *20*, 1563–1571. https://doi.org/10.1016/j.celrep.2017.07.064.

Chelikani, V., Ranjan, T., Zade, A., Shukla, A., and Kondabagil, K. (2014). Genome segregation and packaging machinery in Acanthamoeba polyphaga mimivirus is reminiscent of bacterial apparatus. J. Virol. *88*, 6069–6075. https://doi.org/10.1128/JVI.03199-13.

Chemla, Y.R., Aathavan, K., Michaelis, J., Grimes, S., Jardine, P.J., Anderson, D.L., and Bustamante, C. (2005). Mechanism of force generation of a viral DNA packaging motor. Cell *122*, 683–692. https://doi.org/10.1016/j.cell.2005.06.024.

Chevreuil, M., Law-Hine, D., Chen, J., Bressanelli, S., Combet, S., Constantin, D., Degrouard, J., Möller, J., Zeghal, M., and Tresset, G. (2018). Nonequilibrium self-assembly dynamics of icosahedral viral capsids packaging genome or polyelectrolyte. Nat. Commun. *9*, 1–9. https://doi.org/10.1038/s41467-018-05426-8.

Christo-Foroux, E., Alempic, J.-M., Lartigue, A., Santini, S., Labadie, K., Legendre, M., Abergel, C., and Claverie, J.-M. (2020). Characterization of Mollivirus kamchatka, the first modern representative of the proposed Molliviridae family of giant viruses. J. Virol. *94*. e01997–19. https://doi.org/10.1128/JVI.01997-19.

Colson, P., De Lamballerie, X., Yutin, N., Asgari, S., Bigot, Y., Bideshi, D.K., Cheng, X.-W., Federici, B.A., Van Etten, J.L., and Koonin, E.V.((2013). '"Megavirales", a proposed new order for eukaryotic nucleocytoplasmic large DNA viruses'. Arch. Virol. *158*, 2517–2521. https://doi.org/10.1007/s00705-013-1768-6.

CellPress
OPEN ACCESS

Colson, P., and Raoult, D. (2010). Gene repertoire of amoeba-associated giant viruses. Intervirology *53*, 330–343. https://doi.org/10.1159/000312918.

Colson, P., La Scola, B., and Raoult, D. (2017). Giant viruses of amoebae: a journey through innovative research and paradigm changes. Annu. Rev. Virol. *4*, 61–85. https://doi.org/10.1146/annurev-virology-101416-041816.

Counterman, A.E., and Clemmer, D.E. (1999). Volumes of individual amino acid residues in gas-phase peptide ions. J. Am. Chem. Soc. *121*, 4031–4039. https://doi.org/10.1021/ja984344p.

Cui, J., Schlub, T.E., and Holmes, E.C. (2014). An allometric relationship between the genome length and virion volume of viruses. J. Virol. *88*, 6403–6410. https://doi.org/10.1128/JVI.00362-14.

Desnues, C., La Scola, B., Yutin, N., Fournous, G., Robert, C., Azza, S., Jardot, P., Monteil, S., Campocasso, A., Koonin, E.V., et al. (2012). Provirophages and transpovirons as the diverse mobilome of giant viruses. Proc. Natl. Acad. Sci. U S A *109*, 18078–18083. https://doi.org/10.1073/pnas.1208835109.

Donelli, G. (1968). Isolation of a bacteriophage of exceptional dimensions active in R. megatherium. Atti Accad Naz Lincei-Rend Clas Sci. Fis Mat Nat. *44*, 95, (In Italian.).

Donelli, G., Dore, E., Frontali, C., and Grandolfo, M.E. (1975). Structure and physico-chemical properties of bacteriophage G: III. A homogeneous DNA of molecular weight 5× 108. J. Mol. Biol. *94*, 555–565. https://doi.org/10.1016/0022-2836(75)90321-6.

Dragulescu, A.A., and Arendt, C. (2012). Xlsx: Read, Write, Format Excel 2007 and Excel 97. 2000/XP/2003 Files. https://CRAN.R-project.org/package=xlsx.

Edwards, K.F., Steward, G.F., and Schvarcz, C.R. (2021). Making sense of virus size and the tradeoffs shaping viral fitness. Ecol. Lett. *24*, 363–373. https://doi.org/10.1111/ele.13630.

Effantin, G., Hamasaki, R., Kawasaki, T., Bacia, M., Moriscot, C., Weissenhorn, W., Yamada, T., and Schoehn, G. (2013). Cryo-electron microscopy three-dimensional structure of the jumbo phage ΦRSL1 infecting the phytopathogen ralstonia solanacearum. Structure *21*, 298–305. https://doi.org/10.1016/j.str.2012.12.017.

Van Etten, J.L. (2003). Unusual life style of giant chlorella viruses. Annu. Rev. Genet. *37*, 153–195. https://doi.org/10.1146/annurev.genet.37.110801.143915.

Van Etten, J.L., Lane, L.C., and Dunigan, D.D. (2010). DNA viruses: the really big ones (giruses). Annu. Rev. Microbiol. *64*, 83–99. https://doi.org/10.1146/annurev.micro.112408.134338.

Van Etten, J.L., Lane, L.C., and Meints, R.H. (1991). Viruses and viruslike particles of eukaryotic algae. Microbiol. Rev. *55*, 586–620.

Evilevitch, A., Lavelle, L., Knobler, C.M., Raspaud, E., and Gelbart, W.M. (2003). Osmotic pressure inhibition of DNA ejection from phage. Proc. Natl. Acad. Sci. U S A *100*, 9292–9295. https://doi.org/10.1073/pnas.1233721100.

Filee, J. (2013). Route of NCLDV evolution: the genomic accordion. Curr. Opin. Virol. *3*, 595–599. https://doi.org/10.1016/j.coviro.2013.07.003.

Finsterbusch, T., and Mankertz, A. (2009). 'Porcine circoviruses—small but powerful'. Virus Res. *143*, 177–183. https://doi.org/10.1016/j.virusres.2009.02.009.

Forterre, P. (2017). Viruses in the 21st century: from the curiosity-driven discovery of giant viruses to new concepts and definition of life. Clin. Infect. Dis. *65*, S74–S79. https://doi.org/10.1093/cid/cix349.

Goldhill, D.H., and Turner, P.E. (2014). The evolution of life history trade-offs in viruses. Curr. Opin. Virol. *8*, 79–84. https://doi.org/10.1016/j.coviro.2014.07.005.

Grayson, P., Evilevitch, A., Inamdar, M.M., Purohit, P.K., Gelbart, W.M., Knobler, C.M., and Phillips, R. (2006). The effect of genome length on ejection forces in bacteriophage lambda. Virology *348*, 430–436. https://doi.org/10.1016/j.virol.2006.01.003.

Guo, P., Peterson, C., and Anderson, D. (1987). Prohead and DNA-gp3-dependent ATPase activity of the DNA packaging protein gp16 of bacteriophage ϕ29. J. Mol. Biol. *197*, 229–236. https://doi.org/10.1016/0022-2836(87)90121-5.

Hingamp, P., Grimsley, N., Acinas, S.G., Clerissi, C., Subirana, L., Poulain, J., Ferrera, I., Sarmento, H., Villar, E., and Lima-Mendez, G. (2013). Exploring nucleo-cytoplasmic large DNA viruses in Tara Oceans microbial metagenomes. ISME J. *7*, 1678–1695. https://doi.org/10.1038/ismej.2013.59.

Hu, Y., Zandi, R., Anavitarte, A., Knobler, C.M., and Gelbart, W.M. (2008). Packaging of a polymer by a viral capsid: the interplay between polymer length and capsid size. Biophysical J. *94*, 1428–1436. https://doi.org/10.1529/biophysj.107.117473.

Hua, J., Huet, A., Lopez, C.A., Toropova, K., Pope, W.H., Duda, R.L., Hendrix, R.W., and Conway, J.F. (2017). Capsids and genomes of jumbo-sized bacteriophages reveal the evolutionary reach of the HK97 fold. mBio *8*, 1–15. https://doi.org/10.1128/mBio.01579-17.

Hulo, C., De Castro, E., Masson, P., Bougueleret, L., Bairoch, A., Xenarios, I., and Le Mercier, P. (2011). ViralZone: a knowledge resource to understand virus diversity. Nucleic Acids Res. *39*, D576–D582. https://doi.org/10.1093/nar/gkq901.

Iyer, L.M., Aravind, L., and Koonin, E.V.((2001). Common origin of four diverse families of large eukaryotic DNA viruses. J. Virol. *75*, 11720–11734. https://doi.org/10.1128/JVI.75.23.11720-11734.2001.

Jover, L.F., Effler, T.C., Buchan, A., Wilhelm, S.W., and Weitz, J.S. (2014). The elemental composition of virus particles: implications for marine biogeochemical cycles. Nat. Rev. Microbiol. *12*, 519–528. https://doi.org/10.1038/nrmicro3289.

King, A.M.Q., Lefkowitz, E., Adams, M.J., and Carstens, E.B. (2011). Virus Taxonomy: Ninth Report of the International Committee on Taxonomy of Viruses (Elsevier).

Klug, W.S., Roos, W.H., and Wuite, G.J.L. (2012). Unlocking internal prestress from protein nanoshells. Phys. Rev. Lett. *109*, 168104. https://doi.org/10.1103/PhysRevLett.109.168104.

Koonin, E.V., Dolja, V.V., Krupovic, M., Varsani, A., Wolf, Y.I., Yutin, N., Zerbini, F.M., and Kuhn, J.H. (2020). global organization and proposed megataxonomy of the virus world. Microbiol. Mol. Biol. Rev. *84*. e00061–19. https://doi.org/10.1128/MMBR.00061-19.

Koonin, E.V., and Starokadomskyy, P. (2016). Are viruses alive? The replicator paradigm sheds decisive light on an old but misguided question. Stud. Hist. Philos. Sci. C: Stud. Hist. Philos. Biol. Biomed. Sci. *59*, 125–134. https://doi.org/10.1016/j.shpsc.2016.02.016.

Korn, E.D., and Weisman, R.A. (1967). Phagocytosis of latex beads by acanthamoeba: II. Electron microscopic study of the initial events. J. Cell Biol. *34*, 219–227. https://doi.org/10.1083/jcb.34.1.219.

Legendre, M., Bartoli, J., Shmakova, L., Jeudy, S., Labadie, K., Adrait, A., Lescot, M., Poirot, O., Bertaux, L., and Bruley, C. (2014). Thirty-thousand-year-old distant relative of giant icosahedral DNA viruses with a pandoravirus morphology. Proc. Natl. Acad. Sci. U S A *111*, 4274–4279. https://doi.org/10.1073/pnas.1320670111.

Legendre, M., Lartigue, A., Bertaux, L., Jeudy, S., Bartoli, J., Lescot, M., Alempic, J.-M., Ramus, C., Bruley, C., and Labadie, K. (2015). In-depth study of Mollivirus sibericum, a new 30,000-y-old giant virus infecting Acanthamoeba. Proc. Natl. Acad. Sci. U S A *112*, E5327–E5335. https://doi.org/10.1073/pnas.1510795112.

Legendre, M., Fabre, E., Poirot, O., Jeudy, S., Lartigue, A., Alempic, J.M., Beucher, L., Philippe, N., Bertaux, L., and Christo-Foroux, E. (2018). Diversity and evolution of the emerging Pandoraviridae family. Nat. Commun. *9*, 1–12. https://doi.org/10.1038/s41467-018-04698-4.

Legendre, M., Alempic, J.-M., Philippe, N., Lartigue, A., Jeudy, S., Poirot, O., Ta, N.T., Nin, S., Couté, Y., and Abergel, C. (2019). Pandoravirus celtis illustrates the microevolution processes at work in the giant Pandoraviridae genomes. Front. Microbiol. *10*, 430. https://doi.org/10.3389/fmicb.2019.00430.

Li, Y., Endo, H., Gotoh, Y., Watai, H., Ogawa, N., Blanc-Mathieu, R., Yoshida, T., and Ogata, H. (2019). 'The earth is small for "Leviathans": long distance dispersal of giant viruses across aquatic environments'. Microbes Environ. *34*, 334–339. https://doi.org/10.1264/jsme2.ME19037.

Lynch, M., and Marinov, G.K. (2015). The bioenergetic costs of a gene. Proc. Natl. Acad. Sci. U S A *112*, 15690–15695. https://doi.org/10.1073/pnas.1514974112.

Mahmoudabadi, G., Milo, R., and Phillips, R. (2017). The energetic cost of building a virus. Proc. Natl. Acad. Sci. U S A *114*, E4324–E4333. https://doi.org/10.1073/pnas.1701670114.

Mihara, T., Nishimura, Y., Shimizu, Y., Nishiyama, H., Yoshikawa, G., Uehara, H., Hingamp, P., Goto, S., and Ogata, H. (2016). Linking virus genomes with host taxonomy. Viruses *8*, 66. https://doi.org/10.3390/v8030066.

Milo, R., Jorgensen, P., Moran, U., Weber, G., and Springer, M. (2010). 'BioNumbers—the database of key numbers in molecular and cell biology'. Nucleic Acids Res. *38*, D750–D753. https://doi.org/10.1093/nar/gkp889.

Monier, A., Claverie, J.-M., and Ogata, H. (2008). Taxonomic distribution of large DNA viruses in the sea. Genome Biol. *9*, 1–15. https://doi.org/10.1186/gb-2008-9-7-r106.

Morita, M., Tasaka, M., and Fujisawa, H. (1993). DNA packaging ATPase of bacteriophage T3. Virology *193*, 748–752. https://doi.org/10.1006/viro.1993.1183.

Nurmemmedov, E., Castelnovo, M., Catalano, C.E., and Evilevitch, A. (2007). Biophysics of viral infectivity: Matching genome length with capsid size. Q. Rev. Biophys. *40*, 327–356. https://doi.org/10.1017/S0033583508004666.

Okamoto, K., Miyazaki, N., Reddy, H.K.N., Hantke, M.F., Maia, F.R.N.C., Larsson, D.S.D., Abergel, C., Claverie, J.-M., Hajdu, J., Murata, K., et al. (2018). Cryo-EM structure of a Marseilleviridae virus particle reveals a large internal microassembly. Virology *516*, 239–245. https://doi.org/10.1016/j.virol.2018.01.021.

De Paepe, M., and Taddei, F. (2006). 'Viruses' life history: towards a mechanistic basis of a trade-off between survival and reproduction among phages'. PLoS Biol. *4*, 1248–1256. https://doi.org/10.1371/journal.pbio.0040193.

Perlmutter, J.D., Perkett, M.R., and Hagan, M.F. (2014). Pathways for virus assembly around nucleic acids. J. Mol. Biol. *426*, 3148–3165. https://doi.org/10.1016/j.jmb.2014.07.004.

Perlmutter, J.D., Qiao, C., and Hagan, M.F. (2013). Viral genome structures are optimal for capsid assembly. Elife *2*, e00632. https://doi.org/10.7554/eLife.00632.

Philippe, N., Legendre, M., Doutre, G., Couté, Y., Poirot, O., Lescot, M., Arslan, D., Seltzer, V., Bertaux, L., Bruley, C., et al. (2013). Pandoraviruses: amoeba viruses with genomes up to 2.5 Mb reaching that of parasitic eukaryotes. Science *341*, 281–286. https://doi.org/10.1126/science.1239181.

Phillips, R., Kondev, J., Theriot, J., and Garcia, H. (2012). Physical biology of the cell (Garland Scieme).

Purohit, P.K., Inamdar, M.M., Grayson, P.D., Squires, T.M., Kondev, J., and Phillips, R. (2005). Forces during bacteriophage DNA packaging and ejection. Biophysical J. *88*, 851–866. https://doi.org/10.1529/biophysj.104.047134.

Rao, V.B., and Feiss, M. (2008). The bacteriophage DNA packaging motor. Annu. Rev. Genet. *42*, 647–681. https://doi.org/10.1146/annurev.genet.42.110807.091545.

Raoult, D., Audic, S., Robert, C., Abergel, C., Renesto, P., Ogata, H., La Scola, B., Suzan, M., and Claverie, J.-M. (2004). The 1.2-megabase genome sequence of Mimivirus. Science *306*, 1344–1350. https://doi.org/10.1126/science.1101485.

Raoult, D., and Boyer, M. (2010). Amoebae as genitors and reservoirs of giant viruses. Intervirology *53*, 321–329. https://doi.org/10.1159/000312917.

Rau, D.C., Lee, B., and Parsegian, V.A. (1984). Measurement of the repulsive force between polyelectrolyte molecules in ionic solution: hydration forces between parallel DNA double helices. Proc. Natl. Acad. Sci. *81*, 2621–2625. https://doi.org/10.1073/pnas.81.9.2621.

Reteno, D.G., Benamar, S., Khalil, J.B., Andreani, J., Armstrong, N., Klose, T., Rossmann, M., Colson, P., Raoult, D., and La Scola, B. (2015). Faustovirus, an asfarvirus-related new lineage of giant viruses infecting amoebae. J. Virol. *89*, 6585–6594. https://doi.org/10.1128/JVI.00115-15.

Rodrigues, R.A.L., Abrahão, J.S., Drumond, B.P., and Kroon, E.G. (2016). Giants among larges: how gigantism impacts giant virus entry into amoebae. Curr. Opin. Microbiol. *31*, 88–93. https://doi.org/10.1016/j.mib.2016.03.009.

Saberi, A., Gulyaeva, A.A., Brubacher, J.L., Newmark, P.A., and Gorbalenya, A.E. (2018). A planarian nidovirus expands the limits of RNA genome size. PLoS Pathog. *14*, e1007314. https://doi.org/10.1371/journal.ppat.1007314.

Schoch, C.L., Ciufo, S., Domrachev, M., Hotton, C.L., Kannan, S., Khovanskaya, R., Leipe, D., Mcveigh, R., O'Neill, K., and Robbertse, B. (2020). NCBI Taxonomy: a comprehensive update on curation, resources and tools. Database *2020*, baaa062. https://doi.org/10.1093/database/baaa062.

Sharma, R., Pielstick, B.A., Bell, K.A., Nieman, T.B., Stubbs, O.A., Yeates, E.L., Baltrus, D.A., and Grose, J.H. (2019). A novel, highly-related jumbo family of bacteriophages that were isolated against Erwinia. Front. Microbiol. *10*, 1533. https://doi.org/10.3389/fmicb.2019.01533.

Shukla, A., Chatterjee, A., and Kondabagil, K. (2018). The number of genes encoding repeat domain-containing proteins positively correlates with genome size in amoebal giant viruses. Virus Evol. *4*, vex039. https://doi.org/10.1093/ve/vex039.

Slimani, M., Pagnier, I., Raoult, D., and La Scola, B. (2013). Amoebae as battlefields for bacteria, giant viruses, and virophages. J. Virol. *87*, 4783–4785. https://doi.org/10.1128/JVI.02948-12.

Subramaniam, K., Behringer, D.C., Bojko, J., Yutin, N., Clark, A.S., Bateman, K.S., van Aerle, R., Bass, D., Kerr, R.C., and Koonin, E.V., (2020). A new family of DNA viruses causing disease in crustaceans from diverse aquatic biomes. MBio *11*. .e02938–19. https://doi.org/10.1128/mBio.02938-19.

Sun, S., Kondabagil, K., Draper, B., Alam, T.I., Bowman, V.D., Zhang, Z., Hegde, S., Fokine, A., Rossmann, M.G., and Rao, V.B. (2008). The structure of the phage T4 DNA packaging motor suggests a mechanism dependent on electrostatic forces. Cell *135*, 1251–1262.

Team, R.C. (2020). A Language and Environment for Statistical Computing (R Foundation for Statistical Computing). https://www.R-project.org/.

Thomas, R., Grimsley, N., Escande, M., Subirana, L., Derelle, E., and Moreau, H. (2011). Acquisition and maintenance of resistance to viruses in eukaryotic phytoplankton populations. Environ. Microbiol. *13*, 1412–1420. https://doi.org/10.1111/j.1462-2920.2011.02441.x.

Tzlil, S., Kindt, J.T., Gelbart, W.M., and Ben-Shaul, A. (2003). Forces and pressures in DNA packaging and release from viral capsids. Biophysical J. *84*, 1616–1627. https://doi.org/10.1016/S0006-3495(03)74971-6.

Valbuena, A., Maity, S., Mateu, M.G., and Roos, W.H. (2020). Visualization of single molecules building a viral capsid protein lattice through Stochastic pathways. ACS Nano *14*, 8724–8734. https://doi.org/10.1021/acsnano.0c03207.

Wellehan, J.F.X., Lierz, M., Phalen, D., Raidal, S., Styles, D.K., Crosta, L., Melillo, A., Schnitzer, P., Lennox, A., Lumeij, J.T., et al. (2016). Infectious disease. Current Therapy in Avian Medicine and Surgery (Elsevier), pp. 22–106.

Wickham, H. (2016). GGPLOT2: Elegant Graphics for Data Analysis (2016) (Springer-Verlag). https://ggplot2.tidyverse.org.

Wickham, H., Francois, R., Henry, L., and Müller, K. (2015). 'dplyr: A Grammar of Data Manipulation', R Package Version 0.4, 3. https://CRAN.R-project.org/package=dplyr.

Woese, C.R., Kandler, O., and Wheelis, M.L. (1990). Towards a natural system of organisms: proposal for the domains Archaea, Bacteria, and Eucarya. Proc. Natl. Acad. Sci. U S A *87*, 4576–4579. https://doi.org/10.1073/pnas.87.12.4576.

Xiao, C., Fischer, M.G., Bolotaulo, D.M., Ulloa-Rondeau, N., Avila, G.A., and Suttle, C.A. (2017). Cryo-EM reconstruction of the Cafeteria roenbergensis virus capsid suggests novel assembly pathway for giant viruses. Sci. Rep. *7*, 5484. https://doi.org/10.1038/s41598-017-05824-w.

Xiao, C., Kuznetsov, Y.G., Sun, S., Hafenstein, S.L., Kostyuchenko, V.A., Chipman, P.R., Suzan-Monti, M., Raoult, D., McPherson, A., and Rossmann, M.G. (2009). Structural Studies of the Giant Mimivirus. PLoS Biology *7*, e1000092. https://doi.org/10.1371/journal. pbio.1000092.

Yoshikawa, G., Blanc-Mathieu, R., Song, C., Kayama, Y., Mochizuki, T., Murata, K., Ogata, H., and Takemura, M. (2019). Medusavirus, a novel large DNA virus discovered from hot spring water. J. Virol. *93*. e02130–18. https://doi.org/10.1128/JVI.02130-18.

Yuan, Y., and Gao, M. (2017). Jumbo bacteriophages: an overview. Front. Microbiol. *8*, 403. https://doi.org/10.3389/fmicb.2017.00403.

Zandi, R., Dragnea, B., Travesset, A., and Podgornik, R. (2020). On virus growth and form. Phys. Rep. *847*, 1–102. https://doi.org/10.1016/j.physrep.2019.12.005.

Zandi, R., and Reguera, D. (2005). Mechanical properties of viral capsids. Phys. Rev. E *72*, 21917. https://doi.org/10.1103/PhysRevE.72.021917.

STAR★METHODS

KEY RESOURCES TABLE

Resource	Source	Identifier
Software and algorithms		
R Project for Statistical Computing	(Team, 2020)	RRID:SCR_001905 https://www.R-project.org/
R Package – ggplot2	(Wickham, 2016)	RRID:SCR_014601
R Package – stats	(Team, 2020)	N/A
R Package – dplyr	(Wickham et al., 2015)	RRID:SCR_016708
R Package – xlsx	(Dragulescu and Arendt, 2012)	N/A
Other		
ViralZone Database	(Hulo et al., 2011)	RRID:SCR_006563 https://viralzone.expasy.org/
NCBI viral genomes resource	(Brister et al., 2015)	RRID:SCR_006472 https://www.ncbi.nlm.nih.gov/

RESOURCE AVAILABILITY

Lead contact

Further information and requests for resources should be directed to and will be fulfilled by the lead contact, Dr. Kiran Kondabagil; Email ID: kirankondabagil@iitb.ac.in, Telephone Number: +91-22-25767758.

Materials availability

This study did not generate any new materials.

Data and code availability

Data sets used for this study are provided in the Table S1. The present research did not use any new codes.

METHOD DETAILS

Data collection

For this study, only viruses that infect all types of life forms were considered. Viruses such as virophages and satellite viruses that rely on other viral infections for their replication cycle were not considered. Data on genome type and length, capsid size, and shape of viruses were collected from ViralZone:https://viralzone.expasy.org/ (Hulo et al., 2011) and NCBI databases:https://www.ncbi.nlm.nih.gov/ (Brister et al., 2015). In the cases of some giant viruses and jumbo phages, data were also gathered from literature (references available in the Table S1). Information regarding the host type and entry mechanism was also collected from the literature. Data on genome sizes obtained from ViralZone were verified with the NCBI database. For viruses with a segmented genome, the total size of all segments was considered. To remove statistical redundancy, viruses with the same capsid and genome sizes were represented only once in the plot. Wherever the capsid size is denoted by a range, an average of the highest and lowest value was taken. Regardless of whether the virus is enveloped or nonenveloped or it has an irregular nucleocapsid, we used the representative values of capsid radius from the ViralZone database. Details of the parameters collected for all viruses including the type of genetic material are shown in the Table S1.

In the case of bacterial viruses, only the capsid head dimensions were used for estimating the capsid volume. The data for capsid size/shape and genome size were meticulously collected from a wide variety of sources. Although the errors in the genome size are expected to be negligible, there may likely be few errors in the measurement of capsid sizes, for example, owing to physiological conditions under which capsid sizes were measured, especially for smaller viruses (Figure S3). Although such discrepancies, in themselves, are small enough to be inconsequential, they make their presence felt in a few cases (around 5 of 85 dsDNA

icosahedral bacteriophages) where we find that the packing density of DNA in the viral capsids (as explained below in the section "Volume and packaging density calculation") turns out to be greater than one, something that is not possible unless the double helix of the DNA could itself be distorted during packaging. Hence, we remove such isolated cases (5 bacterial viruses) from our data set as their packaging ratio is greater than 1, which might be owing to errors in capsid dimensions. These are, however, isolated cases and are not expected to cause any significant modifications in scaling exponents.

Our final data set consisted of a total of 381 viruses – 114 bacterial, 69 plant, 100 vertebrate (52 human), 29 invertebrate, 11 algae, 32 protozoan, 8 fungal, and 18 archaeal viruses. The bacterial viruses considered included 29 jumbo phages.

QUANTIFICATION AND STATISTICAL ANALYSIS

Volume and packaging density calculation

In the case of icosahedral viruses, particle size was approximated from the capsid dimensions. For nonicosahedral viruses, capsid volume was estimated using the particle size parameters, namely, length, breadth, and height. The volume of capsid was calculated using different formulas for different shapes, spherical (icosahedral) viruses $\left(V = \frac{4}{3}\pi R_c^3\right)$, Ovoid (lemon-shaped) viruses $\left(V = \frac{4}{3}\pi a^2 c\right)$, filamentous (rod) viruses ($V = \pi R_c^2 l$), and brick viruses ($V = hdl$). In these formulas, V is the virion volume, R_c is the capsid outer radius, a is the equatorial radius of the spheroid, c is the distance from center to pole along the symmetry axis, l is virion length, h is height, and d is depth. These formulas were also used in a previous allometry study (Cui et al., 2014).

For further analysis of the dsDNA icosahedral viruses subgroup considered (bacteriophages, jumbo phages, and NCLDVs), the capsid volume was corrected by considering the thickness of the capsid protein wall which was estimated to be about 3 nm in the case of bacteriophages (Božič et al., 2013) and 10 nm in the cases with an internal lipid membrane, such as NCLDVs (Xiao et al., 2017; Okamoto et al., 2018; Yoshikawa et al., 2019). In the case of *Mimivirus*, the capsid is made up of 5 different layers, so the capsid thickness value was taken as 70 nm (Xiao et al., 2009) , and in the case of *Marseillevirus*, the capsid is made up of a single lipid bilayer giving a capsid thickness of about 10 nm (Okamoto et al., 2018). For other NCLDVs, capsid thickness was taken as 10 nm considering that all are having a single lipid bilayer.

Packaging ratio for dsDNA icosahedral viruses was calculated as (V_g/V_c), where V_c is the capsid volume and V_g is the genome volume calculated as ($V_g = 0.34 * \pi * L_{g)}$ by assuming DNA to be a cylinder of radius 1 nm and each base pair is separated by ~0.34 nm (Purohit et al., 2005) and L_g is the genome length in base pairs.

Data analysis

The statistical analysis was performed in R v4.0.3 (Team, 2020). Log-log plots were made using ggplot package. For linear regression fit, *lm* function from stats package was used. The xlsx and dplyr packages were used to read excel sheets and to subgroup the data, respectively.

Following the previous allometry study (Cui et al., 2014), we have classified the data to update the scaling relation between genome size and particle size as per (I) Baltimore classification: dsDNA (n = 197), ssDNA (n = 30), dsRNA (n = 26), ssRNA(+) (n = 88), ssRNA(-) (n = 26), reverse-transcribing (dsDNA [n = 8], ssRNA [n = 6]) (n = 14) (Figures S1A–S1F); (II) enveloped (n = 113) and nonenveloped viruses (n = 268), (Figure S2A & B); and (III) icosahedral viruses (including spherical) (n = 291) and nonicosahedral viruses (includes rod, ovoid, filamentous, prolate, brick) (n = 90) (Figures S2C and S2D). A comprehensive study of scaling relation in these subgroups is provided in the Table S2. For a detailed analysis of the impact of a host on the relationship between particle size and genome size, two subgroups of dsDNA icosahedral viruses, bacterial viruses (n = 80), and viruses infecting unicellular eukaryotes such as protozoan and algae were selected (n = 28).

Calculation of viral energetic cost

In their detailed analysis, Mahmoudabadi et al. (2017) calculated energy costs for making a virus from different viral life-cycle components and reported that genome replication and protein translation costs for capsid are the two most dominant ones. We use a similar approach as theirs to find the energy costs for the bacteriophages and NCLDVs.

Replication energy

Following Mahmoudabadi et al. (2017) the replication energy for the virus can be calculated directly using genome length L_g in base-pairs as

$$E_{REP(dsDNA)/v} = 2(L_g)(e_d + e_p + e_{od}), \quad \text{(Equation 1)}$$

where, the single DNA nucleotide cost is taken as the sum of the average direct cost of DNA synthesis from precursor metabolites (e_d), the cost of chain elongation per base (e_p), and the average opportunity cost per nucleotide (e_{od}) (Mahmoudabadi et al., 2017). Multiplication by factor of 2 arises because of the double-stranded nature of the genome. The reported numerical values of various energy costs in Equation 1 are e_p = 2 ATP (Lynch and Marinov, 2015), e_d = 11 ATP, e_{od} = 34 ATP (bacterial host), and e_{od} = 42 ATP (eukaryote host). Values are taken from (Mahmoudabadi et al., 2017). More details about these issues are provided in the section on metabolic costs. Genome packaging in most large dsDNA viruses is accomplished by an ATP-driven packaging motor (Rao and Feiss, 2008; Sun et al., 2008; Chelikani et al., 2014). The cost of DNA packaging in bacteriophages φ29 and T3 was estimated to be about one ATP for every two base pairs of DNA translocated (Guo et al., 1987; Morita et al., 1993; Chemla et al., 2005). However, as can be seen from Equation 1, the replication cost per base pair of DNA is approximately 94 ATP (bacterial host) and 110 ATP (eukaryote host). Hence, as the DNA packaging cost is significantly lesser as compared to DNA replication energy cost, we do not include it in our calculations.

Similarly, by assuming that the viral capsid of radius R_c is half filled with DNA (Jover et al., 2014), the DNA replication cost for the virus can also be obtained as follows:

$$E_{rep(dsDNA)/v} = \frac{4\pi r_c^3}{3v_d}(e_d + e_p + e_{od}). \quad \text{(Equation 2)}$$

Here, packaged genome length is obtained by dividing capsid inner volume by v_d ($\approx$1 nm^3) (Milo et al., 2010), the approximate volume of a single base pair and r_c is the capsid inner radius.

TRANSLATION ENERGY

Following the study by Mahmoudabadi et al. (2017), we assume that the main cost of protein translation is associated with capsid proteins. Hence, for a virus with inner radius r_c,

$$E_{tl(dsDNA)/v} = \frac{4\pi r_c^2 t}{v_a}(e_a + e_{ea} + e_{oa}), \quad \text{(Equation 3)}$$

where, e_a is the average direct cost to produce amino acids from precursor metabolites, e_{ea} is the cost of formation of each polypeptide bond, and e_{oa} is the average opportunity cost per amino acid. Here, the number of amino acids is roughly obtained by dividing the capsid volume by v_a ($\approx$ 0.1 nm^3) (Counterman and Clemmer, 1999), the approximate volume of an amino acid. The thickness t of the capsid is taken as 3 nm for bacteriophages (Božič et al., 2013) and 10 nm for NCLDVs (Xiao et al., 2017; Okamoto et al., 2018; Yoshikawa et al., 2019). The numerical values for e_{ea} =4 ATP (Phillips et al., 2012), e_a = 2 ATP, e_{oa} = 25 ATP (bacterial host), and e_{oa} = 30 ATP (eukaryotes host) (values are taken from [Mahmoudabadi et al., 2017]). Note that, because the protein copy numbers making up the capsid for most NCDLVs have not yet been elucidated, we cannot get the exact translation cost using actual number of amino acids. Hence, we resort to the approximation made in Equation 3.

iScience, Volume *24*

Supplemental information

Scaling relation between genome length and particle size of viruses provides insights into viral life history

Harshali V. Chaudhari, Mandar M. Inamdar, and Kiran Kondabagil

Supplemental Information

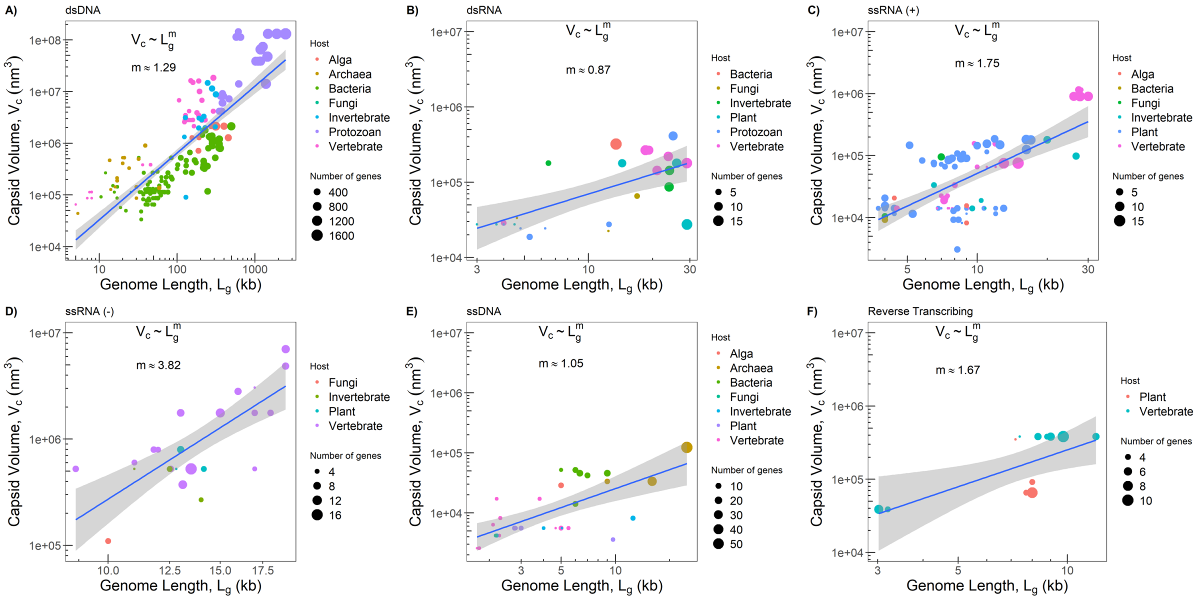

Figure S1. The log-log plot of outer capsid volume as a function of genome length for viruses infecting different hosts according to the Baltimore classification. Related to Figure 1 and STAR Methods "Data analysis".

Size of the data points indicate the number of genes. A power law $y = ax^m$ is used as a fitting expression for the entire data. A linear regression fit of the form $Y = m X + A$ to the data, where $Y = log\, y$, $X = log\, x$, and $A = log\, a$, gives

(A) $m = 1.29$ for dsDNA viruses

(B) $m = 0.87$ for dsRNA viruses

(C) $m = 1.75$ for ssRNA (+) positive sense viruses

(D) $m = 3.82$ for ssRNA (-) negative sense viruses

(E) $m = 1.05$ for ssDNA viruses

(F) $m = 1.67$ for reverse transcribing viruses.

All logs are to the base 10. Data and formulas are presented in the Table S1.

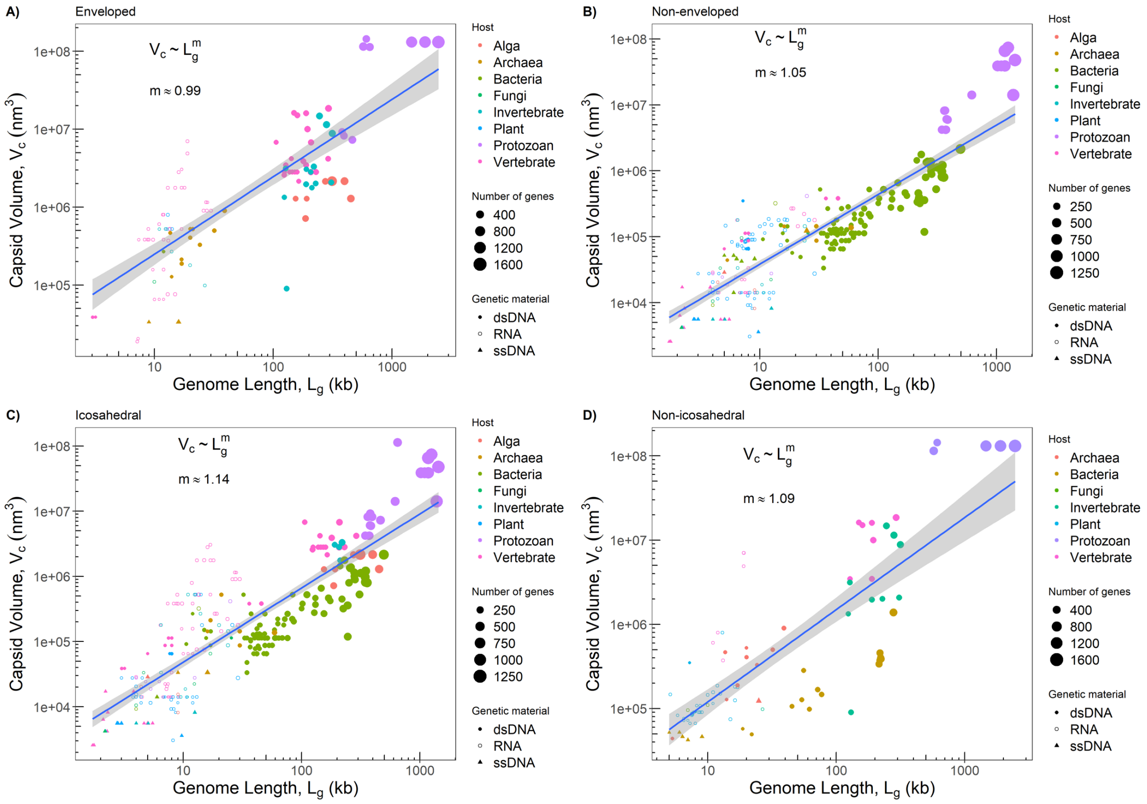

Figure S2. The log-log plot of outer capsid volume as a function of genome length for viruses infecting different hosts according to enveloped (A), non-enveloped (B), icosahedral (C), and non-icosahedral (D) viruses. Related to Figure 1 and STAR Methods "Data analysis".

Size of the data points indicate the number of genes. A power law $y = ax^m$ is used as a fitting expression for the entire data. A linear regression fit of the form $Y = m\,X + A$ to the data, where $Y = log\,y$, $X = log\,x$, and $A = log\,a$, gives

(A) $m = 0.99$ for enveloped viruses

(B) $m = 1.05$ for non-enveloped viruses

(C) $m = 1.14$ for icosahedral viruses

(D) $m = 1.09$ for non-icosahedral viruses.

All logs are to the base 10. Data and formulas are presented in the Table S1.

Packaging Density	Phages	NCLDVs
Median	0.503	0.097
SD	0.458	0.164

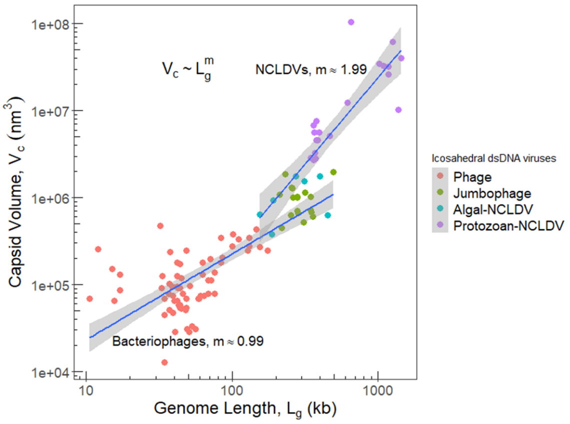

Packaging Density	Phages	NCLDVs
Median	0.454	0.102
SD	0.424	0.131

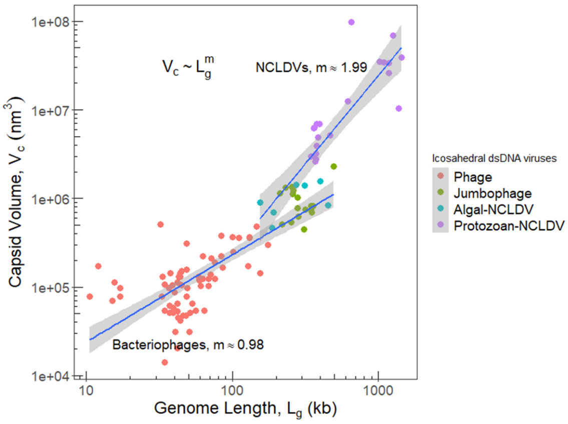

Packaging Density	Phages	NCLDVs
Median	0.461	0.102
SD	0.448	0.129

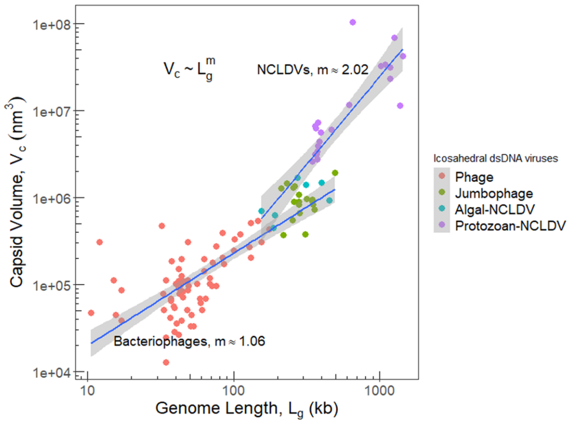

Packaging Density	Phages	NCLDVs
Median	0.496	0.097
SD	0.368	0.167

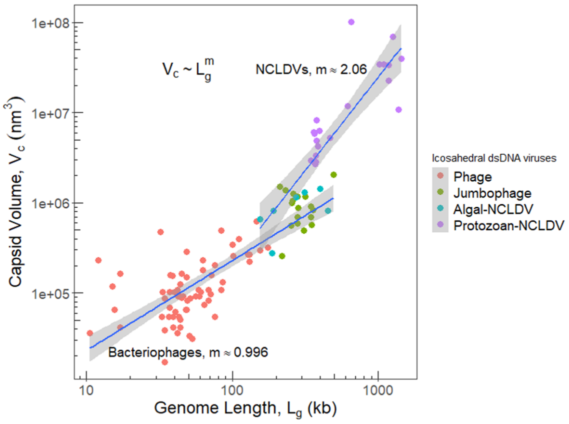

Figure S3. The log-log plot of inner capsid volume versus genome length for icosahedral dsDNA bacteriophages (including jumbophages) and NCLDVs (algal and protozoan). Related to STAR Methods "Data collection".

Inner radius estimated by subtracting capsid thickness, 3 nm for bacteriophages and 10 nm for NCLDVs, from the outer radius. The influence on the scaling relations and median packaging density of error ΔD in the capsid diameter that is randomly sampled from $[-10\text{ nm}, +10\text{ nm}]$ is shown for four separate instances. A small error $\Delta R = \Delta D/2$ in the measurement/reporting of capsid size R could give a larger error in packing density for small R as in the equation below: $\rho_{\text{exact}} = \frac{L_g}{4/3\pi R^3(1+\Delta R/R)^3} \approx \frac{L_g}{4/3\pi R^3}(1 - 3\Delta R/R) = \rho_{\text{calculated}}(1 - 3\Delta R/R)$. As can be seen from this simple calculation, the error in capsid measurement ΔR has more severe implications on density measurement for smaller R.

Table S2. Power law fit, $y = ax^m$, for capsid volume (*y*) and genome length (*x*) for group of viruses classified according to classification based on

(I) Baltimore classification

(II) Enveloped and non-enveloped

(III) Icosahedral and non-icosahedral viruses.

Related to Figure1 and STAR Methods "Data analysis".

Allometric exponents m and the associated statistical parameters (R^2 and p) for linear regression of the form $Y = mX + A$, where $Y = log\, y$ and $X = log\, x$, and $A = log\, a$ to the data for different hosts. All logs are to the base 10.

Classification	No of viruses	*m*	*A*	R^2	p-value
Baltimore classification					
dsDNA	197	1.29	3.23	0.66	$< 2.2\times10^{-16}$
ssDNA	30	1.05	3.35	0.43	5.033×10^{-05}
dsRNA	26	0.87	3.97	0.39	0.0004
ssRNA (+)	88	1.75	2.96	0.42	7.236×10^{-12}
ssRNA (-)	26	3.82	1.62	0.56	6.561×10^{-06}
Reverse transcribing	14	1.67	3.73	0.38	0.01122
Enveloped and non-enveloped viruses					
Enveloped	113	0.99	4.40	0.63	$< 2.2\times10^{-16}$
Non-enveloped	268	1.05	3.53	0.76	$< 2.2\times10^{-16}$
Icosahedral and non-icosahedral viruses					
Icosahedral	291	1.14	3.55	0.69	$< 2.2\times10^{-16}$
Non-icosahedral	90	1.09	3.99	0.64	$< 2.2\times10^{-16}$
